# Macrophage Reprogramming via the Modulation of Unfolded Protein Response with siRNA-Loaded Magnetic Nanoparticles in a TAM-like Experimental Model

**DOI:** 10.3390/pharmaceutics15061711

**Published:** 2023-06-12

**Authors:** Annarita D’Urso, Francesca Oltolina, Chiara Borsotti, Maria Prat, Donato Colangelo, Antonia Follenzi

**Affiliations:** Department of Health Sciences, School Medicine, Università del Piemonte Orientale A. Avogadro, Via Solaroli 17, 28100 Novara, Italy; annarita.durso@uniupo.it (A.D.); francesca.oltolina@med.uniupo.it (F.O.); chiara.borsotti@uniupo.it (C.B.);

**Keywords:** tumor-associated macrophages, macrophage polarization, UPR response, magnetic nanoparticles, siRNA

## Abstract

New therapeutic strategies are required in cancer therapy. Considering the prominent role of tumor-associated macrophages (TAMs) in the development and progression of cancer, the re-education of TAMs in the tumor microenvironment (TME) could represent a potential approach for cancer immunotherapy. TAMs display an irregular unfolded protein response (UPR) in their endoplasmic reticulum (ER) to endure environmental stress and ensure anti-cancer immunity. Therefore, nanotechnology could be an attractive tool to modulate the UPR in TAMs, providing an alternative strategy for TAM-targeted repolarization therapy. Herein, we developed and tested polydopamine-coupled magnetite nanoparticles (PDA-MNPs) functionalized with small interfering RNAs (siRNA) to downregulate the protein kinase R (PKR)-like ER kinase (PERK) expression in TAM-like macrophages derived from murine peritoneal exudate (PEMs). After the evaluation of the cytocompatibility, the cellular uptake, and the gene silencing efficiency of PDA-MNPs/siPERK in PEMs, we analyzed their ability to re-polarize in vitro these macrophages from M2 to the M1 inflammatory anti-tumor phenotype. Our results indicate that PDA-MNPs, with their magnetic and immunomodulator features, are cytocompatible and able to re-educate TAMs toward the M1 phenotype by PERK inhibition, a UPR effector contributing to TAM metabolic adaptation. These findings can provide a novel strategy for the development of new tumor immunotherapies in vivo.

## 1. Introduction

The past decade has witnessed fast developments and achievements in cancer immunotherapy, opening new frontiers to cure this pathology [1], which, with its severe morbidity and mortality worldwide, is still referred to as the modern disease per excellence [2]. In immunotherapy, the agents are designed to direct patients’ innate or adaptive immune systems against cancer cells and to remove the cancer immunosuppression that sustains tumor progression [3]. Despite excellent results obtained with immune checkpoint inhibitors (ICIs), especially in hematological malignancies [4], lymphomas [5], and skin cancer [6], cancer immunotherapies still reveal major limitations, such as off-target effects, autoimmunity, and nonspecific inflammatory responses [7]. In addition, the efficacy of immunotherapy in some solid tumors remains far less than expected [8]. Most of the targets that have been chosen as receptors for immunotherapies are related to cancer cell biology or cancer stem cells, with little consideration of the tumor microenvironment (TME), which actually represents a major obstacle limiting therapeutic effectiveness [9]. Recently, cancer research has been significantly focusing on the understanding of the TME in order to provide a basis for new therapeutic strategies that might enhance antitumor immunity or avoid drug resistance to conventional therapies. In the TME, cells represent a dynamic network where cancer cells and several immune cells interact with each other and modulate tumorigenesis and progressive development [10,11]. Among the TME immune cells, tumor-associated macrophages (TAMs) are the most abundant (up to 50% in some solid tumors), and increased TAM infiltration is often associated with poor prognosis [12,13]. Thus, TAMs are attractive targets for cancer immunotherapy [14,15]. Based on morphological, phenotypical, and functional heterogeneity, macrophages are distinguished into two subtypes: classically activated macrophages (M1) and alternatively activated macrophages (M2). M1 macrophages play a crucial role in antitumor immunity and primarily mediate proinflammatory processes, while M2 macrophages have protumor and anti-inflammatory features. Macrophages oscillate between these two phenotypes to respond to and accommodate different physiological conditions. These phenotypic changes are often referred to as “polarization” [16]. Under the influence of cancer cell-derived signals, TAMs mainly exhibit an M2-like phenotype, which promotes the formation of blood and lymphatic vessels, enhances tumor cell proliferation and migration, and induces immunosuppression [17,18]. Therefore, the reprogramming of TAMs from the M2 to the M1 phenotype may represent a breakthrough for cancer immunotherapy [19,20]. Nevertheless, it is important to underline that the M1/M2 classification of macrophages, as proposed in the early 21st century, is a simplified approach and should be used with caution in in vivo studies, where the landscape is far more complex; at the same time, it represents a good model for in vitro studies [16]. In the TME, the environmental stress imposed on TAMs is high and leads to an increase in intracellular energetic demands, such as high need of new proteins, provoking a burden to the endoplasmic reticulum (ER). Indeed, it is widely reported that TAMs display an irregular unfolded protein response (UPR) in their ER, an adaptive signaling pathway aiming at restoring ER homeostasis [21]. In addition, recent studies have suggested that two UPR signal activators, Protein kinase R (PKR)-like ER kinase (PERK) and Inositol-requiring protein 1α (IRE1α), contribute to the metabolic adaptation of TAMs in opposite ways: the PERK arm of the UPR is a critical metabolic hub for the immunosuppressive function of TAMs (M2 phenotype), while the activity of IRE-1α could be decreased in TAMs [22,23]. Indeed, the overactivation of protein IRE1α-mediated X-box-binding protein (XBP1) signaling promotes a M1 phenotype and is involved in the pathogenesis of several inflammatory diseases, as reported by numerous studies [24]. For this reason, it is becoming increasingly clear that the modulation of UPR in macrophages could be a promising strategy to re-educate TAMs in the TME, as well as a potential therapeutic approach for autoimmune diseases. Nanomedicine is a very exciting field of cancer research. Indeed, in oncology, the use of nanomaterials (1–200 nm), whose behavior can be described with neither classical physics nor quantum mechanics, has often been designated over the years as an evolution of the “magic bullet” concept, since they could constitute suitable platforms to develop the next generation of programmable and personalized nanomedicine [25]. In this scenario, the convergence of immunotherapy and nanotechnology is surely generating substantial momentum for improving cancer treatment [26,27]. Among a large variety of nanomaterials with both therapeutic and diagnostic applications, iron oxide nanoparticles (MNPs), such as magnetite crystals (Fe_3_O_4_), have received considerable interest as nanocarriers for targeted drug delivery to enhance and ensure the high specificity of immunotherapies [28]. Indeed, MNPs can be multi-functionalized and manipulated by an external gradient magnetic field (GMF) in order to deliver different classes of biologically active molecules, such as antiproliferative drugs, antibodies, and lentiviral vectors [29,30,31,32,33]. Moreover, they can also be employed as agents to induce hyperthermia when subjected to alternating magnetic fields (AMF) [34]. Strikingly, MNPs might also serve as immune modulators by mimicking or enhancing immune cell functions [35]. Many studies have shown that the iron oxide core of these nanoplatforms has strong effects on macrophage polarization toward the M1 phenotype, probably by affecting signaling pathways linked to the iron metabolism or the production of reactive oxygen species, thus altering the intracellular redox balance [36]. Nevertheless, the immune response induced by MNPs is limited, but appropriate modifications on the surface of MNPs could allow for the combination of all desired properties of these nanoplatforms into one single nanosystem to obtain promising tools for selective targeting of TAMs. In recent years, polydopamine (PDA), a versatile biomaterial derived from the self-polymerization of dopamine, has gained great attention as an agent used for surface modifications of different nanomaterials [37]. Due to its similar structure to melanin and its unique adhesion properties, PDA has sparked considerable interest in photodynamic therapy (PDT), antibacterial applications, theranostics, and tissue repair [38]. Furthermore, PDA has abundant functional groups on the surface (i.e., amine and catechol), which could bind various biomolecules, including single-stranded DNA and small interfering RNA (siRNA) [39]. siRNAs have been reported as potential therapeutic agents for gene therapy due to their involvement in endogenous post-transcriptional regulation, known as the RNA interference (RNAi) mechanism [40]. Based on this evidence, we propose, for the first time, to re-educate in vitro pro-tumorigenic M2-like macrophages into anti-tumor M1-like macrophages by modulating UPR with siRNA-loaded magnetic nanocarriers. In particular, we developed PDA-MNPs functionalized with siPERK and tested them in macrophages derived from murine peritoneal exudate (PEMs). PEMs were differentiated in vitro and characterized towards either the M1 or the M2 phenotype. Finally, we evaluated the ability of PDA-MNPs/siPERK to re-educate TAM-like macrophages from the M2 to the M1 phenotype.

## 2. Materials and Methods

### 2.1. Preparation and Characterization of PDA-MNPs Nanoparticles

Purely inorganic magnetite nanoparticles (Fe_3_O_4_), here reported as MNPs, were provided as a sterile water solution (Sigma-Aldrich, St. Louis, MO, USA). PDA-MNPs were prepared by coating MNPs with polydopamine (PDA) shells via dopamine (Sigma-Aldrich) solution oxidation according to the method described by Mu [32]. Briefly, 1 mg of MNPs were resuspended into 0.5 mL of Tris-buffered saline solution (TBS) of pH 8.5, containing 1 mg/mL dopamine (DA). After stirring for 3 h at room temperature, the color of the suspension turned dark brown, indicating that the DA molecules were oxidized and self-polymerized on the surface of the MNPs. The PDA-coated MNPs were then recovered by magnetic decantation, washed three times with TBS, resuspended in TBS, and stored at 4 °C until use. The amount of adsorbed DA was calculated from the difference between the concentrations of the DA in solutions before and after adsorption on the MNPs (the so-called supernatant) and measured by UV−vis spectroscopy at a wavelength of 550 nm [41] using a standard curve of known concentrations of DA solutions as the reference. The hydrodynamic diameter of all nanoparticles (NPs), along with ζ-potential measurements, were carried out by dynamic light scattering (DLS) with a Zetasizer Nano ZS analyzer (Malvern Instruments Ltd., Malvern, UK) in aqueous suspensions (~0.5 mg/mL, room temperature) contained in polystyrene vials. For measurements of ζ-potential versus pH, suspensions of NPs were prepared at pH 7.4 using the MPT2 autotitrator with dilute HCl and NaOH solutions (0.25 and 0.1 M, respectively). The stability of the PDA adsorbed on MNPs was monitored for different times up to 7 days by incubating PDA-MNPs at 37 °C. Afterward, the absorbances of the collected supernatants were analyzed by UV–Vis spectroscopy at a wavelength of 550 nm, and the absorbance was compared to a reference solution of PDA. Each sample was analyzed in triplicate, and the experiment was repeated three times. All solutions were prepared with diethylpyrocarbonate-treated water (H_2_O-DEPC).

### 2.2. Obtention of PEMs

Primary macrophages (PEMs) were isolated from male and female BALB/c mice, 12–16 weeks of age. One milliliter of 3% thioglycolate solution was injected into the peritoneal cavity of the mouse, and after 6 days, macrophages derived from murine peritoneal exudate were isolated according to the method described by Rios et al. [42]. Briefly, mice were killed, and 10 mL of complete medium with a few heparin drops was injected into the abdomen. The fluid was withdrawn and centrifuged (1000× *g* rpm for 5 min at 4 °C), and the cell pellet was resuspended in 10 mL of complete medium. Cells were then washed twice by centrifugation in the same conditions. The pellet was resuspended in complete medium, and cells were plated in 6-well plates (2 × 10^6^ cells/well). After 16 h culture at 37 °C, non-adherent cells were removed, adherent cells were washed with phosphate-buffered saline (PBS) of pH 7.2, and a new medium was added. Then, PEMs were maintained in culture in the presence of macrophage colony stimulating factor (rm m-CSF, ImmunoTools GmbH, Friesoythe, Germany) for several days until use. Balb/c mice, about 12–16 weeks old, were housed under standard conditions in a pathogen-free environment. All procedures were approved (Italian Health Ministry Authorization: #370/2019-PR, project DB064.45) and carried out in accordance with the Animal Care and Use Committee of UPO, the European Community Directive for Care, and Italian Laws on Animal Experimentation (Law by Decree 116/92).

### 2.3. Cytocompatibility Tests

#### 2.3.1. Cell Viability Assay

The RAW 264.7 macrophage cell line was purchased from ATCC and grown in Dulbecco modified Eagle’s medium (DMEM) (Sigma-Aldrich) supplemented with 10% fetal calf serum (FCS), antibiotic solution (streptomycin 100 μg/mL and penicillin 100 U/mL, Sigma-Aldrich), and 2 mM L-glutamine (complete medium) in a humidified atmosphere containing 5% CO_2_ at 37 °C. RAW 264.7 and PEMs (1 × 10^4^ and 2 × 10^4^ cells/microwell of 96-well plates, respectively) were seeded for 24 h and incubated for 72 h with different concentrations of nanoparticles, ranging from 0.1 to 100 μg/mL, which were added in 100 μL of fresh medium. At the end of the incubation time, cell viability was evaluated by an MTT (Sigma-Aldrich) colorimetric assay. Briefly, 20 μL of MTT solution (5 mg/mL in a PBS solution) was added to each well. The plate was then incubated at 37 °C for 2–3 h. After the removal of the solution, 125 μL of isopropanol and 0.2 N HCl were added to dissolve the formazan crystals. Then, 100 μL was then carefully removed, and the optical density was measured in a multi-well reader (2030 Multilabel Reader Victor TM X4, PerkinElmer, Waltham, MA, USA) at 570 nm. The experiments were carried out in triplicate at least three times.

#### 2.3.2. Detection of Reactive Oxygen Species (ROS) Production

To measure the potential oxidative stress in living cells, due to the presence of PDA-MNPs, the CellROX Green Reagent (Thermo Fisher Scientific, Waltham, MA, USA) was used following the protocol recommended by the manufacturer. Briefly, cells (approximately 6 × 10^4^ RAW264.7/well and 4 × 10^5^ PEMs/well) were seeded on glass coverslips in 24-well plates. After cells were incubated with 100 μg/mL of PDA-MNPs for different times (from 4 h to 24 h), cells were washed with PBS, and CellROX Green Reagent was added to a final concentration of 5 μM in 300 μL of DMEM medium without serum, and the plate was incubated in the dark at 37 °C for 30 min. The combination of bacterial lipopolysaccharides (LPS; Sigma-Aldrich) plus interferon gamma (IFN-γ, Immunotools) was used as a positive control. At the end of the incubation, the coverslips were washed with PBS of pH 7.2, fixed with 4% paraformaldehyde (PAF, Thermo Fisher Scientific) in PBS, washed again, and permeabilized with 0.1% Triton-X100 (Sigma-Aldrich) for 10 min. Finally, the coverslips were stained and mounted on specimen slides (Biosigma, Cona, Italy). The cytoskeletal actin was stained with tetramethylrhodamine (TRITC)-phalloidine (1/200, Sigma-Aldrich; excitation at 543 nm; emission at 560–620 nm), and the cell nuclei were stained with DAPI (Thermo Fisher Scientific, 1:50). The CellROX Green Reagent is only fluorescent in the oxidized state because of ROS production. Therefore, the emission of green fluorescence (at 485/520 nm) is stable and is produced after DNA binding; thus, its signal is mainly located in the nucleus. Fluorescence was detected using a fluorescence microscope (Spectral Confocal Leica TCS SP2 AOBS), and images were taken at 200× magnification. ImageJ software 1.5.3 version was used for the analysis.

#### 2.3.3. Detection of ER Stress and the Activation of Unfolded Protein Response (UPR)

The stress of endoplasmic reticulum (ER) caused into cells by PDA-MNPs was detected indirectly by measuring the levels of specific UPR factors. Briefly, cells (approximately 3 × 10^5^ RAW 264.7/well and 2 × 10^6^ PEMs/well) were seeded in 6-well plates and after 24 h they were incubated with 100 μg/mL of PDA-MNPs for different times (from 4 h to 24 h). Tunicamycin (2 μg/mL, Sigma-Aldrich) was used as a positive control. The transcriptional expression of UPR proteins (Bip, XBP1s, ATF4, ATF6) was evaluated by q-RT-PCR, as described below.

### 2.4. Internalization of PDA-MNPs in Cells in the Absence/Presence of a GMF

#### 2.4.1. Prussian Blue Staining

Cells (approximately 6 × 10^4^ RAW 264.7/well and 4 × 10^5^ PEMs/well) were seeded on glass coverslips in 24-well plates, and after 24 h, 100 μg/mL PDA-MNPs were added. After incubating at 37 °C for different times (from 0.5 to 30 min) in the absence (−GMF) and presence (+GMF) of a gradient magnetic field (Mag0201, Nanoeast, Nanjing, China). The coverslips were washed with fresh PBS, pH 7.2, and fixed with PAF (2% in PBS). Then, the Prussian blue solution (1:1 of 2% potassium ferrocyanide and 2% HCl, both in H_2_O) was added to the coverslips. In these conditions, any ferric ion (+3) present in the samples combines with the ferrocyanide and results in the formation of bright blue pigments called Prussian blue or ferric ferrocyanide. After two other washes with fresh PBS, Nuclear Fast Red (Sigma-Aldrich) was added to stain cell nuclei. Finally, the coverslips were washed with H_2_O and mounted on slides by using one drop of Eukitt quick-hardening mounting medium for each sample. The interaction of the stained PDA-MNPs with cells was analyzed by optical microscopy at 100×. Experiments were performed in triplicate at least 3 times.

#### 2.4.2. Iron Quantification by Potassium Thiocyanate

RAW 264.7 and PEMs were seeded in 6-well plates, and after 24 h, incubation at 37 °C, 100 μg/mL PDA-MNPs suspensions in complete DMEM medium were added for different durations (0.5, 5, and 30 min) in the presence and absence of a GMF. At the end of the treatments, supernatants were removed, cells were washed with fresh PBS, trypsinized, transferred to 0.5 mL Eppendorf tubes, and centrifuged at 1000× *g* rpm for 5 min. Then, the cell pellets were dissolved in 37% HCl, mixed with 10% H_2_O_2_, and incubated for 20 min at room temperature. Samples were then reacted with 1 mL of 1% potassium thiocyanate in Milli-Q water, and their absorbance was measured at 490 nm. The concentration of ferric ions in the samples was calculated, referring to the absorbance obtained from a standard curve calculated from known amounts of PDA-MNPs, following the same protocol as Oltolina et al. [34]. The endogenous iron of the cells was subtracted from the treated samples normalized by the untreated control cells. Experiments were performed in triplicate at least 3 times.

#### 2.4.3. Uptake of PDA-MNPs in PEMs

Cells (8 × 10^5^ PEMs/well) seeded in a 12-well plate in complete medium were incubated after 24 h with 20 μg/mL of PDA-MNPs for 30 min in the presence/absence of a GMF and then incubated for a further 24 h. Cells were then harvested after trypsinization, centrifuged, and fixed in 2% PAF in PBS with FBS (20%) and NaN_3_ (0.02%), and then flow cytometry analysis was performed to assess the physical parameters (side-scatter and forward scatter) of cells. Data were analyzed by FlowJo™ v10 Software (Bioscience, Cambridge, MA, USA).

### 2.5. Functionalization of PDA-MNPs with siRNA Molecules

PDA-MNPs were functionalized with small interfering RNA (siRNA) molecules, and the binding capacity of the nanoparticles to siRNA was analyzed by agarose gel electrophoresis. Briefly, different amounts of MNPs and PDA-MNPs (5, 20, and 50 μg in a final volume of 50 μL) were incubated with 10 μM of non-targeting siRNA-MOCK (sc-37007; Santa Cruz Biotechnology, Heidelberg, Germany) under ultrasonic stirring for 30 min at 4 °C. Nanoparticles/siRNA were then separated from their supernatants by magnetic decantation, nanocomplexes were resuspended in 50 μL of DEPC-water, and 10 μL of each sample was run into a 3% *w*/*v* agarose gel electrophoresis in tris-acetate-EDTA running buffer (TAE 1X) at 120 V for 20 min. Additionally, the supernatants were analyzed with this method. siRNA bands were visualized by staining with SYBR. Images were captured under UV illumination, and the densitometric analysis was performed using ImageJ software 1.5.3 version. The amount of siRNA absorbed on PDA-MNPs was calculated in another series of experiments using the fluorescent dye Alexa 488/siMOCK (sc-3890, Santa Cruz Biotechnology). In detail, PDA-MNPs (100 μg/mL) were mixed with 50 nM of siRNA, sonicated for 30 min at 4 °C, and the fluorescence intensity of PDA-MNP-coupled siMOCK was measured by multiwell reader fluorescence spectrophotometry (excitation 490 nm and emission 530 nm; 2030 Multilabel Reader Victor TM X4, PerkinElmer). The concentration of siMOCK on PDA-MNPs was determined using a reference calibration curve obtained with standard solutions of siMOCK at concentrations ranging from 12 to 70 nM. To quantify the siRNA release from nanoparticles, PDA-MNPS/si-MOCK were resuspended in DMEM containing 10% fetal calf serum (FCS), then shaken at 37 °C for different periods of time up to 72 h in an Eppendorf tube. Supernatants were recovered and analyzed by UV–Vis spectroscopy, as described above. The amounts of released siMOCK were referred to as a percentage of the amounts that were initially adsorbed. For biological assays, PDA-MNPs/siRNA nanocomplexes were made by mixing 20 μg of nanoparticles with 100 pmol of siRNA diluted in 50 μL of Opti-MEM Reduced Serum Medium (Thermo Fisher Scientific) and sonicating at 4 °C for 30 min. PDA-MNPs/siRNA were added to the cells for 30 min under the magnetic field and then under normal conditions for 24–72 h before being analyzed. As a positive control, PEMs were also transiently transfected with 100 pmol siRNA using the Lipofectamine 2000 transfection reagent (Invitrogen Life Technologies, Monza, Italy) at a 2:1 (lipid/siRNA) ratio, following the manufacturer’s instructions for siRNA transfection. In this case, lipocomplexes were added to cells for 6 h at 37 °C before the transfection medium was replaced with complete DMEM.

### 2.6. Cellular Uptake of the PDA-MNPs/siRNA

PEMs were incubated with Alexa488/siMOCK either coupled to PDA-MNPs or administered through Lipofectamine 2000 on a glass coverslip in 24-well plates for fluorescence microscopy and directly in plates for flow cytometry analysis. All the samples, including the controls PDA-MNPs and lipocomplexes, were incubated for 24 h, before being analyzed for the uptaken fluorescence and, after being detached by trypsin, by flow cytometry, which was analyzed with the Attune flow cytometer (Invitrogen Life Technologies). For each group, the fluorescence (Ex/Em: 480/520 nm) of Alexa 488/siMOCK per 1 × 10^5^ cells was acquired. The untreated cells were used as a control. Data were analyzed by FlowJo™ v10 Software (Bioscience).

### 2.7. Gene Silencing Efficiency of PERK Protein via PDA-MNPs/siPERK

siPERK duplex (Cat. sc-36214, Santa Cruz Biotechnology) was purchased as a solution of three specific siRNAs. PEMs seeded in a 6-well plate were incubated with siPERK, either coupled to PDA-MNPs or administered through Lipofectamine 2000, and then +/− tunicamycin (TM) for 8 h. Cells were harvested after 24 h and 48 h to analyze both the transcriptional and translational regulation of the PERK gene and other genes of UPR by q-RT-PCR and immunoblot analysis, respectively. The list of primers and antibodies used is reported in Table 1 and Table 2.

### 2.8. In Vitro Polarization of PEMs

PEMs were incubated for 5 days with 5 ng/mL of m-CSF, as described by Hamidzadeh et al. [43]. Next, the cells were cultured for 48 h in complete DMEM supplemented with 20 ng/mL IL-4 (Immunotools) to polarize towards the M2 phenotype, or with 100 ng/mL LPS and 5 ng/mL IFN-γ to polarize towards the M1 phenotype [44]. To obtain TAM-like macrophages, in this study, PEMs were pretreated with IL-4 to induce an M2 phenotype, and then treated for 8 h with the ER stressor TM (2 µg/mL). The expression of genes, cell surface markers, and the activation of molecular pathways associated with two macrophage phenotypes were investigated by different analyses (see below) to confirm that the treatments with the cytokines induced the two phenotypes, as well as the ability of PDA-MNPs/siPERK to reprogram the TAM-like macrophage towards the M1 phenotype.

### 2.9. Quantitative Real-Time PCR

The mRNA levels of genes were analyzed by quantitative real-time PCR (q-RT-PCR). Total RNA was extracted with Trizol (Invitrogen Life Technologies). The RNA concentration and quality were determined by the NanoDrop 2000C spectrophotometer (Thermo Fisher Scientific, Wilmington, DE, USA). After RNA purification and treatment with DNAse I (Thermo Scientific), 1 μg was retrotranscribed in cDNA with the RevertAidTM H Minus First Strand cDNA Synthesis Kit (Thermo Fisher Scientific) using oligo(dT) primers. Gene assays were performed in triplicate for each treatment in a 12 μL reaction volume containing 1 μL of RT products, 6 μL Sso-Fast EVA Green SMX (Bio-Rad, Hercules, CA, USA), and 500 nM of each forward and reverse primer. The sequences of primers are listed in Table 1. An automated CFX96 real-time thermocycler (Bio-Rad) was used, and the reaction conditions were 95 °C for 1 min, followed by 45 cycles at 98 °C for 5 s and an anneal/extend step for 5 s at 60 °C, with data collection. At the end of these cycles, a melting curve (65 °C to 95 °C, with a plate read every 0.5 °C) was performed to assess the specificity of the amplification product by single-peak melting temperature verification. Results were analyzed with Bio-Rad CFX Manager, and the gene expressions were calculated by the ∆∆Ct method, and β-actin served as an internal control.

### 2.10. Immunoblotting

The protein levels were determined by the Western blotting assay. After the different treatments, cells were washed twice in cold PBS and lysed in ice with RIPA Lysis Buffer (20 mM Tris-HCl pH 7.5, 150 mM NaCl, 50 mM HEPES, 0.1% SDS, 1 mM ethylene glycol-bis(2-aminoethylether)-N,N,N′ (EGTA), 1% NP-40, 1% sodium deoxycholate, 2.5 mM sodium pyrophosphate, and 10% glycerol) supplemented with protease inhibitor cocktail (Sigma-Aldrich). Cell lysates were centrifuged at 13,000× *g* rpm at 4 °C for 15 min. Clarified cell extracts (30 μg of protein) were denatured by heating for 5 min at 95 °C in reducing Laemmli buffer; proteins were separated in SDS-PAGE and transferred onto polyvinylidene difluoride (PVDF) filters. Filters were blocked with 5% non-fat dry milk for 2 h, rinsed in water, and probed with different antibodies in TBS of pH 8.0, and 5% BSA overnight at 4 °C. The list of primary antibodies used is reported in Table 2. After extensive washing, immunocomplexes were detected with appropriate horseradish peroxidase-conjugated secondary anti-IgG antibodies (diluted 1:5000, Sigma-Aldrich), followed by enhanced chemiluminescence (ECL kit; Biorad), and analyzed in a Versadoc instrument (Bio-Rad Laboratories S.r.l, Segrate, Milan, Italy). Bands were subjected to densitometric analysis using ImageJ software 1.5.3 version.

### 2.11. Immunofluorescence Microscopy

PEMs were seeded on glass coverslips (12 mm in diameter) in 24-well plates, and, after the different treatments, they were fixed with 4% PAF for 20 min at 25 °C. The cells were washed three times with PBS, permeabilized with TBS-5% BSA-0.1% Triton-X100-5% FCS for 1 h, and then incubated in the dark for 2 h with the primary antibodies reported in Table 3. After three washes with TBS-5% BSA-0.1% Triton-X100, samples were incubated with Alexa Fluor 488-labeled secondary antibodies (1:500, Sigma) and PE-labeled streptavidin (1:500, Sigma-Aldrich), while the cell nuclei were stained with DAPI (1:50, Thermo Fisher Scientific). Finally, coverslips were washed twice in PBS Triton X-100 and mounted with mowiol_4-88 (Sigma-Aldrich). Fluorescence was detected at the fluorescence microscope (Leica DM 2500); in particular, the fluorescence of Alexa Fluor 488 was excited at 488 nm, and the emitted fluorescence was measured at 491–586 nm. PE fluorescence was excited at 488 nm and measured at 575–675 nm. DAPI fluorescence was excited at 405 nm and measured at 420–480 nm. Images were taken at 200× magnification and analyzed by ImageJ software 1.5.3 version.

### 2.12. Flow Cytometry

PEMs were seeded in 12-well plates and underwent the different treatments described. After detachment and centrifugation, cells were incubated in 100 μL of staining buffer (PBS, 20% FBS, and 0.1% NaN_3_) with 20 μL of inactivated normal mouse serum for 30 min at 4 °C to block the Fc receptors on the macrophage plasma membrane before adding the primary antibody, and to prevent non-specific binding. Cells were incubated for a further 30 min in the dark with a single and/or a combination of the antibodies conjugated with single fluorophores listed in Table 4. Then, cells were washed with 1 mL of staining buffer and centrifuged at 1500× *g* rpm for 5 min. The cell pellets were resuspended in 300 μL of FACS buffer, and samples were acquired on the Attune NxT Acoustic Focusing Cytometer (ThermoFisher Scientific); analyses were performed by FlowJo v10^TM^ software (BD Biosciences). The conditions for data acquisition and analysis were established using the unlabeled cells as a negative control. Each experiment was carried out three times, and single representative experiments are displayed. For statistical significance, at least 100,000 cells were analyzed in each sample, and the mean of the fluorescence emitted by these single cells was used.

### 2.13. Statistical Analysis

Data are expressed as the mean ± standard error of at least 3 triplicates. Statistical analyses were performed using one-way ANOVA with a Bonferroni’s multiple comparisons test for grouped analyses using GraphPad Prism version 9.5.1 for Mac, GraphPad Software (GraphPad Prism, San Diego, CA, USA). Statistical differences between the treatments were considered significant when *p* values were *p* < 0.05 (*), *p* < 0.01 (**), *p* < 0.001 (***), and *p* < 0.0001 (****).

## 3. Results and Discussion

### 3.1. Preparation and Characterization of PDA-MNPs

**Figure 1:**
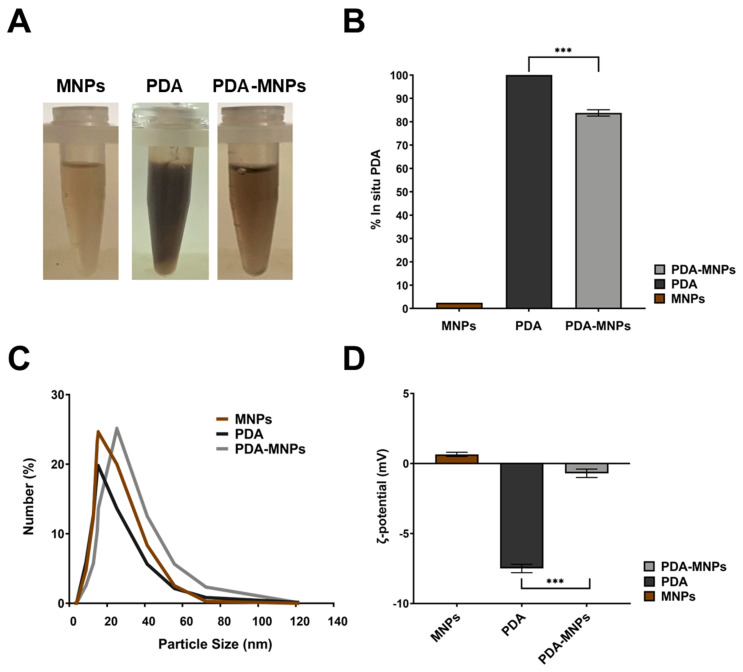
Adsorption of PDA on MNPs. (**A**) Representative images of dopamine after polymerization reaction (PDA) and MNPs before and after coupling (PDA–MNPs). (**B**) Values of PDA (polymerized in situ) measured at 550 nm. The percentage ratio between PDA and PDA–MNPs or MNPs alone is shown. (**C**) Particle size distribution of the prepared PDA-MNPs along with the bare MNPs and PDA, measured by dynamic light scattering (DLS). (**D**) ζ-potential of MNPs, PDA, and PDA–MNPs detected at pH 7.4. The results were obtained from three independent experiments made in triplicate. Differences between groups were assessed by one-way ANOVA with Bonferroni’s multiple comparison post-test (*** *p* ≤ 0.001).

Several surface modifications can be carried out to expand the biomedical applications of MNPs. Versatile biomaterials can be used as coating polymers for MNPs’ functionalization, and among these, polydopamine (PDA), a polymer produced by self-oxidation of dopamine under alkaline conditions, has gained great interest in recent years thanks to its singular adhesive properties similar to those of mussel proteins [37]. In this study, we focused our attention on PDA-functionalized MNP nanocarriers that were prepared by coating the pre-assembled MNPs with PDA shells. Upon incubation with dopamine, MNPs acquired a black color, indicating the adsorption of PDA at their surfaces (Figure 1A). By contrast, the colloidal suspension of MNPs, at the same concentration without PDA coating, remained significantly clearer. The concentration of PDA was quantified by UV–Vis spectroscopy (λ = 550 nm). In particular, after three washes with DEPC-H_2_O, the amount of PDA absorbed on MNPs was 1.2 mg/mg of MNPs, which represents roughly 80% of the originally incubated soluble dopamine (Figure 1B). Moreover, PDA coating on iron oxide nanoparticle surfaces slightly increases the size of the nanoparticles. Indeed, the hydrodynamic diameter, as obtained from DLS measurements, was 30 ± 5 nm in the case of PDA-MNPs, which was larger than that of plain MNPs (15 ± 8 nm) (Figure 1C). Furthermore, the variation of surface charges of uncoated and PDA-coated nanoparticles was investigated at pH 7.4 (physiological human plasma conditions). The results showed that at this pH value, single nanoparticles were negatively charged, thus contributing to their reciprocal repulsion, promoting the stability of the suspension (Figure 1D). In detail, the surface ζ-potential of nanoparticles decreased after PDA coupling at −0.2 mV, suggesting successful coating of MNPs with PDA. The properties of these iron oxide nanoparticles are summarized in Table S1. Moreover, the stability of PDA-MNPs was also tested at different time points (from 1 to 7 days) by evaluating the amount of PDA released from the MNPs in the soluble fraction at 37 °C after they were removed by magnetic decantation. We found that around 98% of the PDA remained stable on the surface for this entire period. In fact, the behavior of PDA coupled to MNPs was similar to the absorbance values of PDA alone at different time points, revealing the integrity of the coating (Figure S1B). Altogether, these data confirm the high stability of these PDA-MNPs at physiological pH.

### 3.2. Isolation and Characterization of Peritoneal Macrophages (PEMs)

**Figure 2:**
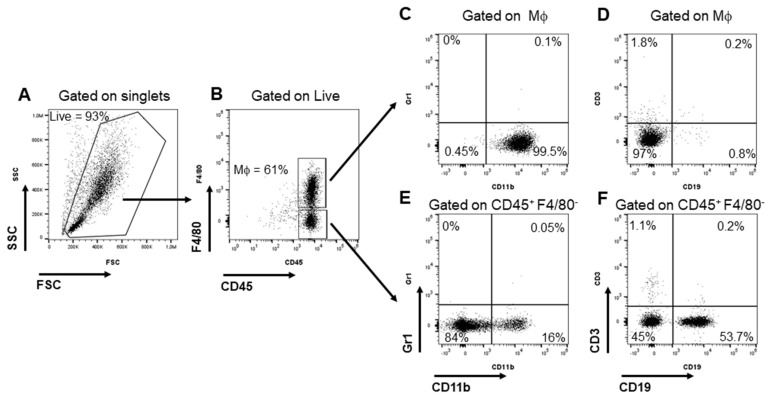
Characterization of primary macrophages from mouse peritoneal exudate (PEMs) by flow cytometry analysis. (**A**) Representative flow cytometry dot plot of the physical parameters of PEMs. FSC: forward scatter (size); SSC: side scatter (granularity). (**B**–**D**) The population of PEMs was characterized using a combination of fluorochrome-conjugated antibodies typical of several immune cell populations: CD45^+^ F4/80^+^ (macrophages, Mϕ), CD11b (myeloid cells), Gr1 (granulocytes), CD19 (B cells), and CD3 (T cells). PEMs were F4/80^+^ CD11b^+^, and negative for Gr1, CD19, and CD3. (**E**,**F**) The CD45^+^ F4/80^−^ population included monocytes and B cells. The results are representative of three independent experiments.

Primary macrophages derived from PEMs were used as experimental in vitro models. They were recovered from BALB/c mice, which received a local administration of thioglycolate to increase their yield from 2–3 to 9–12 million per mouse without altering their physiological characteristics [45]. Physical parameters and some leukocyte markers were analyzed by flow cytometry on the cells collected after 16 h in culture. Despite the presence of several immune cell types, such as B- and T-cells, dendritic cells (DCs), and natural killers (NKs) in the peritoneal cavity [46,47], most of the recovered cells showed a homogenous profile and displayed macrophage phenotypes F4/80^pos^ and CD11b^pos^ (Figure 2A–D). Both CD11b and F4/80 are macrophage markers, but while the CD11b receptor is expressed on the surface of all myeloid cells, glycoprotein F4/80 is expressed exclusively on macrophages. F4/80^pos^ and CD11b^pos^ cells were negative for other blood lineage markers, such as Gr1, CD19, and CD3 (Figure 2C,D). The mouse peritoneal exudate also contained several monocytes and B cells (Figure 2E,F). Overall, these data clearly show that at least 60% of the recovered cells were macrophages.

### 3.3. Cytocompatibility and Cellular Internalization of PDA-MNPs

After confirming macrophage identity, the non-toxicity of PDA-MNPs was ascertained before their possible biomedical application. Thus, we tested the cytocompatibility of PDA-MNPs on two types of murine macrophages: PEMs and the RAW 264.7 cell line.

**Figure 3:**
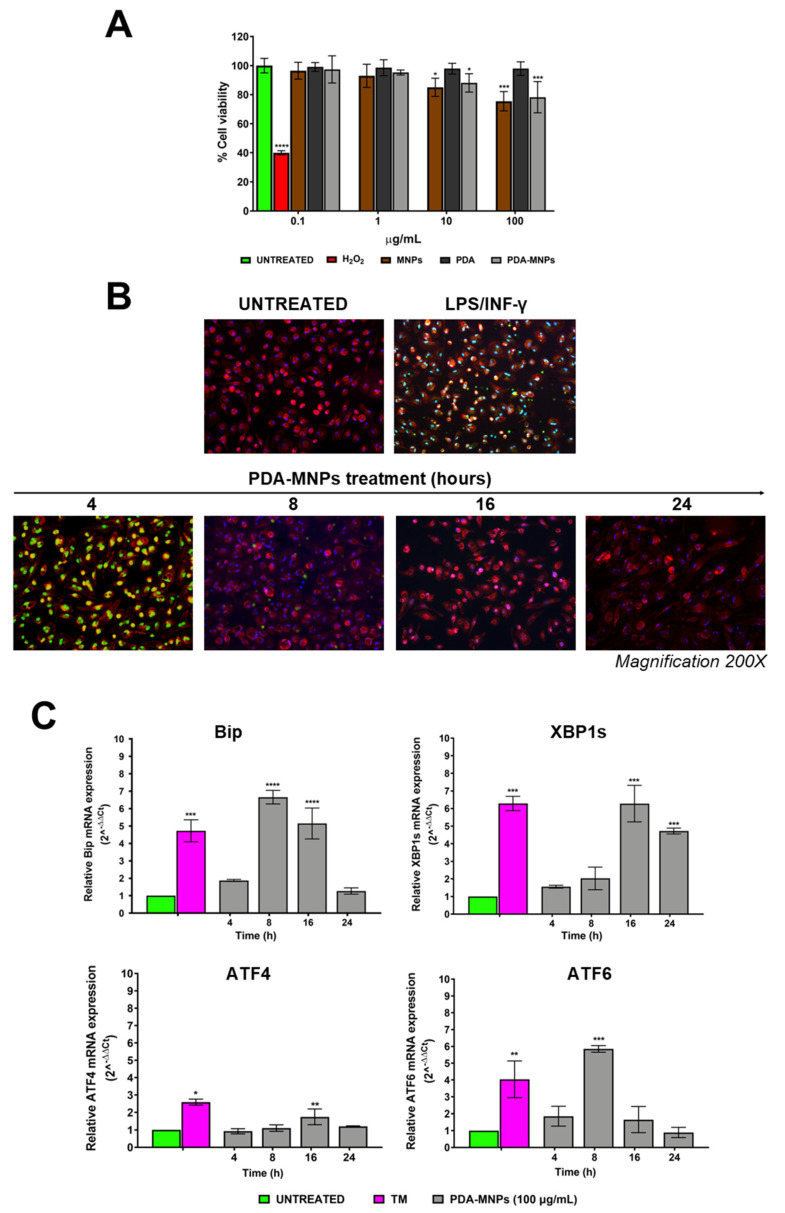
Cytocompatibility of PDA–MNPs on PEM cells. (**A**) Cell viability was assessed by MTT assays after cells were incubated with PDA–MNPs at different concentrations for 72 h. Untreated cells were taken as a reference value (100%), while exposure to H_2_O_2_ at 1 µM represented the positive control. Data are expressed as the mean ± SD of at least four independent experiments performed in triplicate. (**B**) Immunofluorescence images showing reactive oxygen species (ROS) production in PEM cells incubated with PDA–MNPs (100 μg/mL) for different time points (from 4 to 24 h). Fixed and permeabilized cells were stained with TRITC-phalloidin (red) for actin and DAPI (blue) for nuclei, while ROS production was visualized in green. LPS/INF-γ 24 h treated cells were used as a positive control. Magnification, 200×. (**C**) Histograms showing the expression of ER stress markers (Bip, XBP1s, ATF4, and ATF6) assessed by q–RT–PCR on PEM cells incubated with PDA–MNPs (100 μg/mL) in the same conditions as above. ER stress was mostly observed in cells treated with 2 μg/mL tunicamycin for 8 h, used as a positive control. Gene expression is shown as a fold change relative to untreated cells. The data are expressed as the mean ± SD of three independent experiments. Statistical analyses were carried out using one-way ANOVA with the Bonferroni comparison post-test vs. the untreated controls (* *p* ≤ 0.05; ** *p* ≤ 0.01; *** *p* ≤ 0.001; **** *p* < 0.0001).

The viability of both cell lines was assessed in MTT assays after 3 days of incubation. No significant toxicity was observed in RAW 264.7 cells at any nanoparticle concentration (Figure S2A). Indeed, PDA by itself was highly cytocompatible, so the coupling of PDA on MNPs still improved the cytocompatibility of nanocarriers, as also reported in other studies [48,49]. In the case of PEMs, a higher toxicity was observed, especially when cells were incubated with the two highest PDA-MNP concentrations of 10 and 100 μg/mL (Figure 3A), which was not surprising, since primary cells might be more sensitive than immortalized cell lines. Nevertheless, the cell viability of PEMs was always around 80%, which is a value above the cut-off of 70% indicated by ISO 10993-5:2009 [50] as acceptable for clinical applications. Conversely, both macrophage types were sensitive to the addition of hydrogen peroxide (1 μM) used as a positive control since the viability was reduced to 40%. Thus, these data show that PDA-MNPs are endowed with good cytocompatibility on macrophages. Different types of nanoparticles, including those containing graphene, have been reported to induce cytotoxicity by increasing cellular oxidative stress through the generation of reactive oxygen species (ROS) [51]. Therefore, we also analyzed the cytocompatibility of PDA-MNPs by assessing the level of ROS in the two macrophage models. Cells were incubated with the highest dose of PDA-MNPs (100 μg/mL) for different periods of time. Afterwards, the levels of ROS were evaluated by the virtual green color (CellROX^®^ Green Reagent) using fluorescence microscopy. As expected, a noticeable amount of ROS was detected in the positive controls of both cell models stimulated with LPS/INF-γ for 24 h, but also when macrophages were incubated with PDA-MNPs. However, both PEMs (Figure 3B) and RAW 264.7 (Figure S2B) macrophages completely extinguished this response after 4 h and 8 h of incubation with PDA-MNPs, respectively. Since oxidative stress is closely connected with protein-folding homeostasis in the ER [52], we examined whether PDA-MNPs could affect the pathways linked to ER stress. In cells treated with 100 μg/mL of PDA-MNPs, the UPR response was analyzed by quantitative real-time PCR (q-RT-PCR) assessment of the ER chaperone BiP and XBP1s, ATF4, and ATF6 genes. The corresponding proteins are downstream effectors of the ER stress sensors IRE-1, PERK, and ATF6, respectively [53]. We used tunicamycin (TM) as a positive control, since it is a known ER stressor [54]. The results showed that PDA-MNPs were able to activate the UPR response in macrophages. Indeed, in PEMs, ER stress markers were already activated after short incubation with PDA-MNPs, but their gene expression was downregulated after 24 h, except for that of XBP1s (Figure 3C). This finding is in line with the report that XBP1s plays a crucial role in macrophages during inflammatory diseases [24]. Surprisingly, similar behavior was also detected in RAW 264.7 cells (Figure S2C), despite the fact that, since they are a stable cell line, they should be less sensitive to ER stress. Taken together, these results indicate that even if PDA-MNPs affect both cellular redox balance and the normal function of the ER, they do not alter cellular homeostasis, and they are cytocompatible for prolonged exposure time.

**Figure 4:**
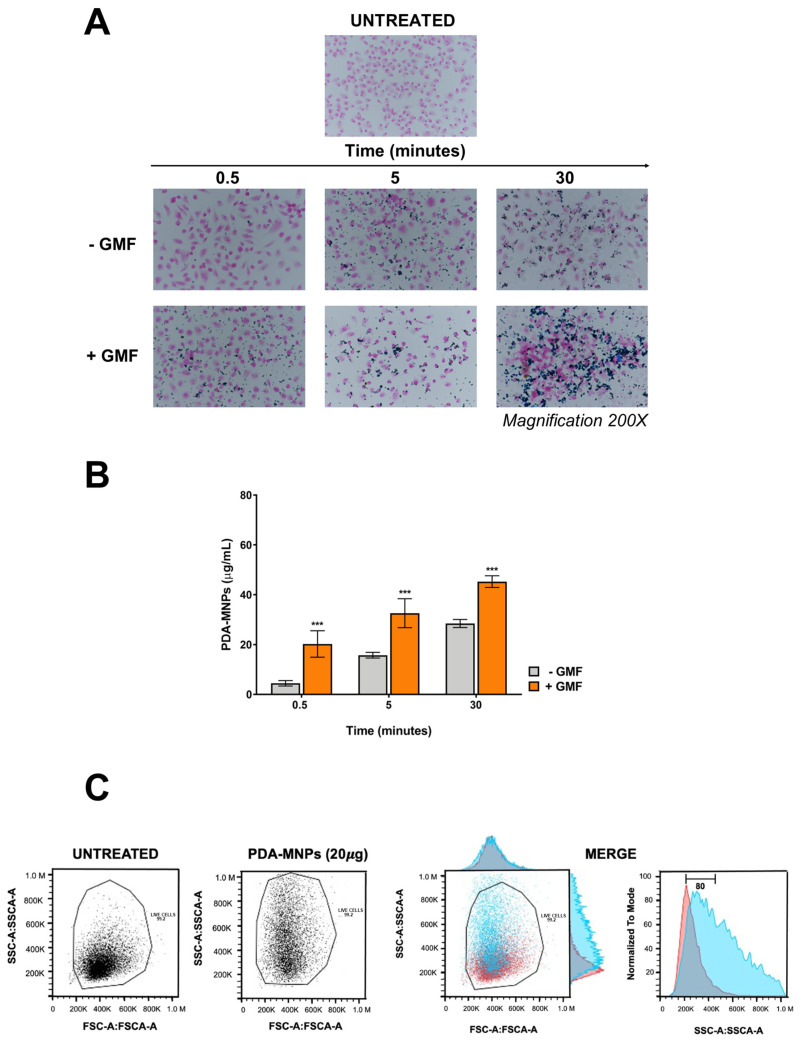
Interaction of PDA–MNPs with PEMs in the presence/absence of a gradient magnetic field (GMF). (**A**) Images show PEMs treated with PDA–MNPs and stained Prussian blue and nuclear fast red. Magnification 200×. (**B**) Amount of iron associated with cells as quantified with potassium thiocyanate: cells were incubated with PDA–MNPs for different time points (from 0.5 to 30 min) in the absence (−GMF) or in the presence (+GMF) of a gradient magnetic field. Untreated cells were used as a negative control. The results are expressed as mean µg/mL ± SD and were obtained from at least three independent experiments performed in triplicate. (**C**) Representative images showing the granularity and size of untreated control cells (left) or cells treated with 20 μg of PDA–MNPs for 24 h. Merge panels are shown on the right and were used for calculations. The data were analyzed by a *t*-test for each time point (*** *p* < 0.001).

It is well known that the application of a gradient magnetic field (GMF) enhances the interaction between magnetic nanoparticles and cells [34]. Thus, RAW 264.7 or PEM macrophages were incubated for different time points with 100 μg/mL PDA-MNPs in the presence or absence of GMF, followed by Prussian blue staining to detect iron oxide nanoparticles. As shown in Figure 4A and Figure S3A, when a magnetic field was applied to both cell types, PDA-MNPs were already clearly visible after 30 s of incubation. By contrast, PDA-MNPs were detectable only after 5 min of incubation in the absence of a magnetic field in both macrophage models. Moreover, PDA-MNPs were increasingly detectable as the incubation time increased in both cell types, even if there was always a significant difference between samples treated with GMF and untreated samples. Similar results were confirmed by the quantification of iron internalized in the cells (Figure 4B and Figure S3B). In fact, in the absence of GMF, the amount of iron associated with macrophages was very low after 30 s of incubation with PDA-MNPs. When the same experiment was performed in the presence of GMF, the iron concentration associated with the cells was already 60 µg/mL for RAW 264.7 and 45 µg/mL for PEMs after 30 s of incubation. This concentration increased in a time-dependent manner until stabilization occurred after 30 min, when there was no significant difference between samples treated with GMF or not, probably because of nanoparticle sedimentation on the cell surface. All together, these data showed that the application of a GMF enhanced the interactions between the nanoparticles and the cells.

We performed a flow cytometry analysis to assess the PDA-MNP internalization into the cells. PEMs interaction with PDA-MNPs affected their granularity, but not their size after 24 h of incubation with a low amount of PDA-MNPs (20 µg/mL) (Figure 4C). In fact, cellular complexity increased by 80% in PDA-MNP-treated PEMs compared to the untreated controls, and this value underwent a further increase in the presence of a GMF. Therefore, we considered the increase in cellular complexity as an indirect measure of the internalization of PDA-MNPs into PEMs.

### 3.4. Gene Silencing Efficiency of siPERK via PDA-MNPs in PEMs

**Figure 5:**
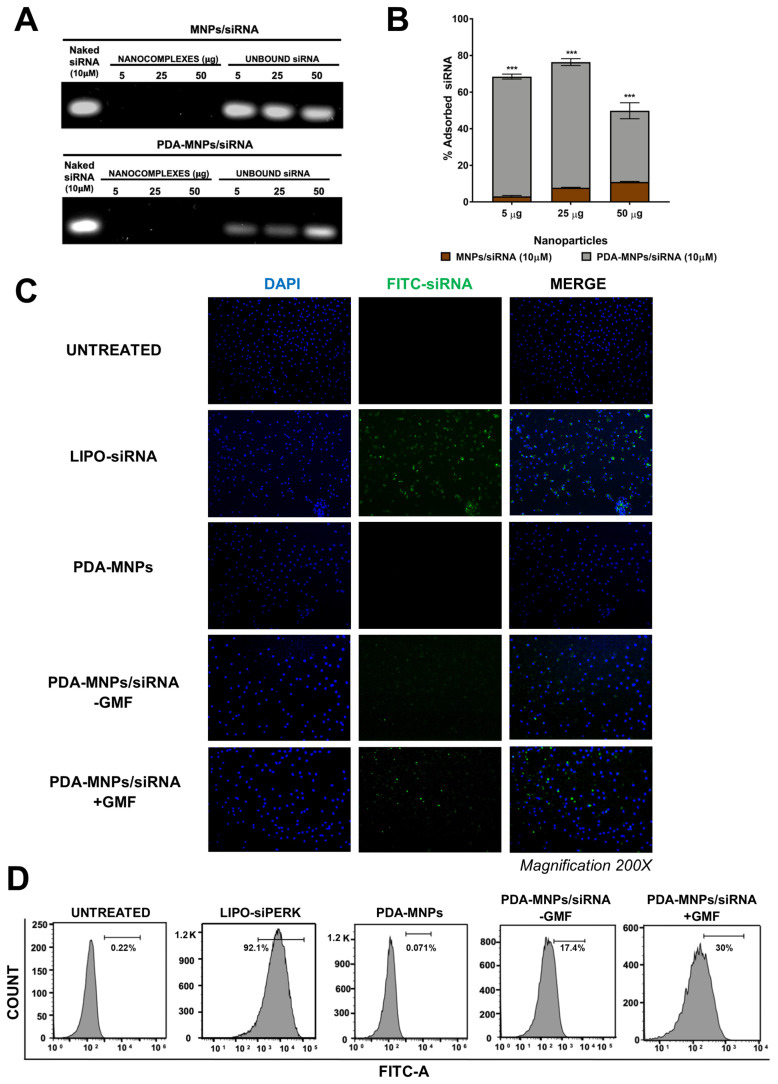
Preparation and the cellular uptake of PDA–MNPs/siRNA in PEM cells. (**A**) Images showing the loading of siRNA onto nanocarriers examined by agarose gel electrophoresis. Different ratios of MNPs and PDA–MNPs with 10 µM siRNA were used in this typical experiment. (**B**) Quantification of the percentage of siRNA bound to MNPs or PDA–MNPs, obtained by subtracting the amount of unbound siRNA from the total siRNA after magnetic decantation (densitometric analysis). The results are expressed as % absorption ± SD of the percentage (delta method); they were compared among them for statistical analysis and were obtained from three independent experiments performed in triplicate. (**C**) Delivery of the siRNAs loaded on nanoparticles into PEMs in the presence/absence of GMF obtained by fluorescence microscopy. Cells were incubated with FITC–labeled siRNA complexes (20 µg) for 24 h. Images were acquired after fixation, permeabilization, and staining for nuclei with DAPI (blue). siRNA transfection with Lipofectamine 2000 (LIPO) and PDA–MNPs alone represent the controls. (**D**) Cell uptake efficiency in PEMs, measured by flow cytometry. The values of FITC–siRNAs are indicated for each treatment. Statistical analyses were performed using a *t*–test for each experimental concentration (*** *p* < 0.001).

Since the PDA polymer surface has numerous groups that are able to facilitate functionalization with biomolecules, PDA-MNPs were designed as magnetic nanocarriers to deliver small interfering RNAs (siRNA) [37]. To assess the feasibility of loading siRNA into nanocarriers, siRNA at a final concentration of 10 μM was incubated with 5, 20, and 50 μg of MNPs or PDA-MNPs under ultrasonic stirring for 30 min and analyzed by agarose gel electrophoresis. As expected, considering that we used a MNPs/siRNA ratio in favor of nanoparticles, nanocomplexes were not able to migrate under the electric field in the agarose gel because of their size (Figure 5A). In addition, the interference of nanoparticles did not allow us to visualize the siRNA associated with these complexes, unlike the plain siRNA used as a control. The capability of NPs to bind siRNA molecules was evaluated by an indirect method: visualization on agarose gel of the amount of unbound siRNA left in the sample after magnetic decantation of the nanoparticles. The amount of siRNA in the soluble fractions obtained after incubation with PDA-MNPs was clearly decreased in a dose-dependent manner, as also confirmed by the densitometric analysis of the bands (Figure 5B). In contrast, in the case of siRNA incubated with naked MNPs, the same original amounts of siRNA were present in the soluble fractions after magnetic decantation of the nanoparticles. These data clearly show that PDA coating is necessary to bind siRNA molecules to MNPs, probably via π–π stacking interactions between the aromatic groups of PDA and the nucleobases of siRNA, as reported in another study [32].

**Figure 6:**
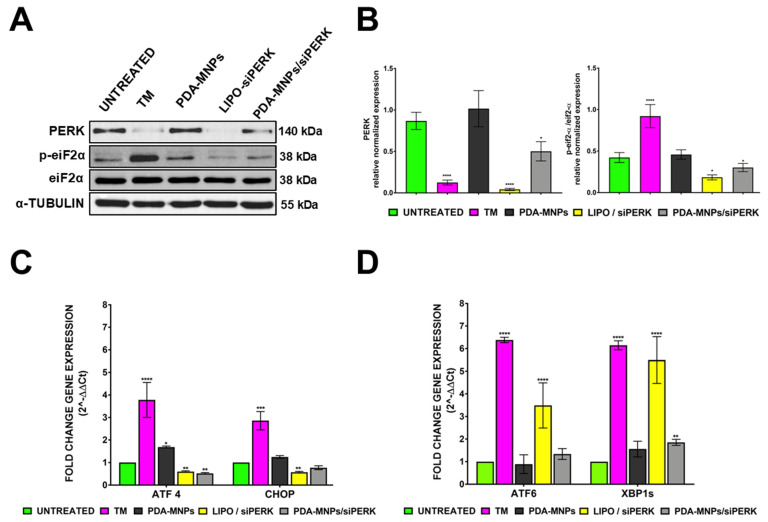
Gene silencing by siRNA delivered by nanoparticles in PEMs. (**A**) WB analysis of the silencing of PERK and its downstream effector phospho–eiF2–α in PEMs treated with LIPO/siPERK and PDAMNPs/siPERK for 48 h. The ER stress inducer tunicamycin (TM, 2 µg/mL) was used as a control along with PDA–MNPs alone. (**B**) Densitometric analysis of protein bands using the protein expression of α-Tubulin as an internal control. (**C**,**D**) Histograms showing mRNA levels of phospho–eiF2–α downstream effectors (ATF4 and CHOP) and ER stress sensors (ATF6 and XBP1) detected by q–RT–PCR following the silencing of PERK in PEM cells with PDA-MNPs/siPERK using LIPO/siPERK as a positive control for gene silencing. Gene expression was expressed as a fold change relative to untreated cells. The data are expressed as the mean ± SD of three independent experiments using one-way ANOVA with the Bonferroni comparison post-test vs. the untreated controls (* *p* ≤ 0.05; ** *p* ≤ 0.01; *** *p* ≤ 0.001; **** *p* < 0.0001).

Afterward, the adsorption efficiency of PDA-MNPs/siRNA was quantified by measuring the fluorescence intensity of a FITC-labeled siRNA (50 nM) after coupling with PDA-MNPs (100 μg/mL). The FITC-siRNA concentration associated with PDA-MNPs after ultrasonic stirring for 30 min was about 70% of the amount found in the original solution. The release of siRNA from PDA-MNPs was also analyzed by evaluating the amount of FITC-siRNA remaining bound to nanoparticles after incubation in complete medium at 37 °C with continuous stirring for different times up to three days. The results showed that PDA-MNPs were able to release the adsorbed siRNA under physiological conditions; in particular, around 50% of the adsorbed siRNA was released after three days (Figure S1B). Since PDA-MNPs are capable of carrying siRNA molecules, we evaluated the ability of these nanocarriers to deliver siRNA to PEMs by fluorescence microscopy. For these experiments, 20 μg of PDA-MNPs was functionalized with a FITC-labeled siRNA (Alexa488/siMOCK), then incubated for 24 h with cells. PEMs in which fluorescent-siRNA was transfected through lipofectamine were used as a positive control. When cells were incubated with PDA-MNPs/siRNA, the green, fluorescent signal was slightly detectable, but when a GMF was applied for 30 min before the 24 h incubation, a significant number of green spots were detectable within the cytoplasm (Figure 5C). Thus, the application of a magnet favored the cellular uptake of PDA-MNPs. As expected, no green signal was detectable when cells were incubated with an equal amount of PDA-MNPs. Furthermore, flow cytometry analysis was also performed to quantify the efficiency of siRNA delivery by PDA-MNPs into PEMs, both in the absence and presence of a GMF. In accordance with the data presented above, primary cells could be efficiently transfected, since more than 92% of the PEMs stained positively (Figure 5D). Instead, only 17% of the cells were able to engulf siRNA complexed with PDA-MNPs in the absence of GMF, but the apposition of GMF enhanced the cellular uptake of siRNA coupled to PDA-MNPs by more than 30%. All together, these results indicate that PDA-MNPs could be promising candidates for delivering siRNA molecules within cells, especially when nanocomplexes are exposed to an external magnetic field. For this reason, we also evaluated the ability of PDA-MNPs/siRNA to induce gene silencing of a target protein, such as PERK ER transmembrane sensor [55]. Therefore, PEMs were either incubated for 24 h or 48 h with siPERK molecules coupled to PDA-MNPs or transfected for the same time with lipocomplexes (LIPO/siPERK), used as a control along with PDA-MNPs alone. After 48 h of treatment with PDA-MNPs/siPERK, PERK protein expression was downregulated in PEM cells, while it was completely silenced in the case of LIPO/siPERK (Figure 6A). Considering that UPR proteins such as PERK are activated in ER stress conditions, we also treated PEM cells in the last 8 h with TM to examine the effect of PERK knockdown on the activation of its downstream signaling pathway. Since the active PERK phosphorylates the downstream mediator eukaryotic translation initiation factor 2 (eif2-α), we also assessed the activation (i.e., the phosphorylation) of this protein. We found that the silencing of PERK significantly reduced the phosphorylation of eif2-α in comparison to the cells treated with TM (Figure 6A,B). A similar behavior was observed when the downregulation of the PERK gene in PEMs was evaluated by analyzing the expression levels of the ATF4 and CHOP genes, downstream effectors of the PERK/eif2-α pathway. In fact, we found that in the presence of ER stress induced by TM, the expression of these two target genes was downregulated in PEM cells because of the incubation with siPERK-containing complexes (Figure 6C). Under ER stress, UPR signal activators act in a coordinated way to detect misfolded proteins in the ER and re-establish cellular homeostasis. Therefore, the activities of IRE-1 and ATF6, other ER stress sensors, along with PERK [56], were examined after the knockdown of PERK.

After 24 h of PERK inhibition with PDA-MNPs/siPERK and LIPO/siPERK, the expression of ATF6 and the spliced form of XBP1, a downstream effector of IRE-1 [53], was analyzed. As reported in Figure 6D, we noticed that PERK inhibition increased the activity of other ER stress sensors compared to untreated cells, but not at the same level as the positive control, which was represented by the TM treatment. In particular, in the case of LIPO/siPERK, a significantly increased splicing of XBP1, involved in macrophage differentiation toward the inflammatory phenotype, was reported by Lara-Reyna et al. [57]. Overall, these results confirm the ability of PDA-MNPs/siPERK to induce PERK target gene silencing in PEMs and to alter the UPR cellular response in which the PERK sensor is involved under ER stress conditions.

### 3.5. In Vitro Differentiation of PEM Macrophages into M1 or M2 Phenotypes

**Figure 7:**
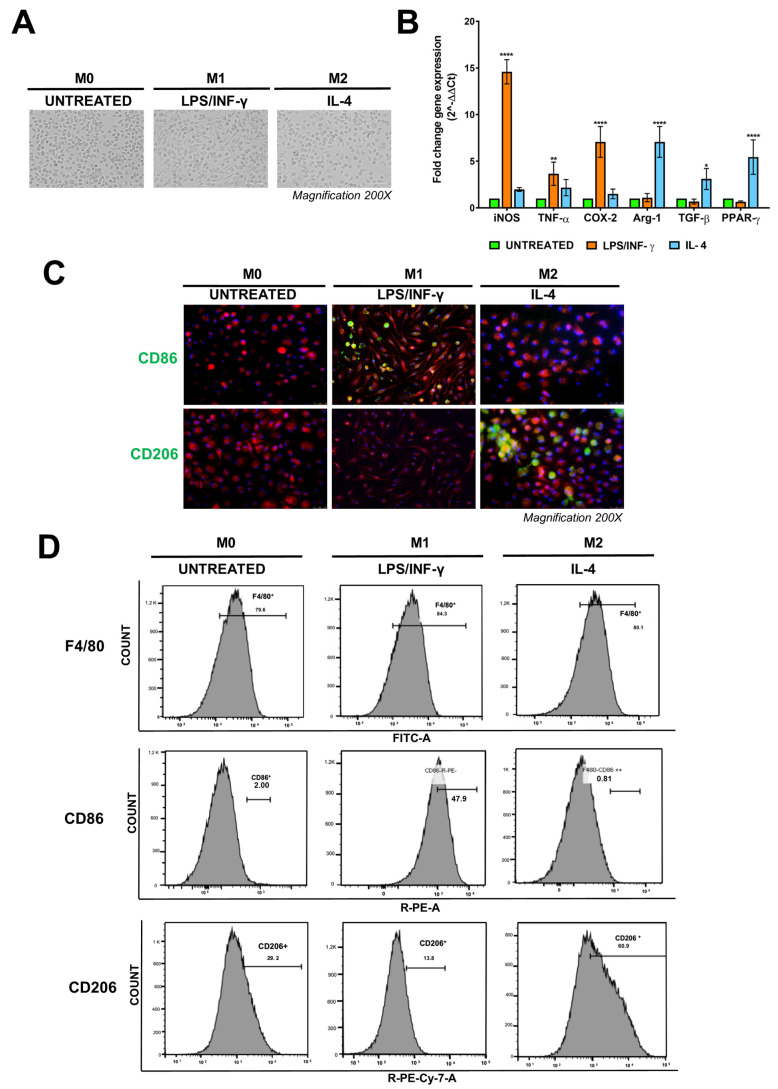
PEMs can be polarized in vitro towards the M1 or M2 phenotypes. (**A**) Images of PEMs after isolation and culture for 5 days with the macrophage colony-stimulating factor (M–CSF) and stimulation for a further 48 h, either with LPS/IFN–γ or IL-4 to induce the M1 and M2 phenotypes, respectively. (**B**) Relative expression of specific genes for M1 and M2 phenotypes was determined by q–RT–PCR. Gene expression was expressed as a fold change relative to untreated M0 cells. The data are expressed as the fold-change expression ± SD of three independent experiments. (**C**) Immunofluorescence images of differentiated PEMs into M1 and M2 phenotypes. Cells were fixed, permeabilized, and stained with FITC-streptavidin F4/80 antibody for macrophage identification (red) and DAPI (blue) for the nuclei. The expression of CD86 (upper lane) and CD206 (lower lane) was visualized by green fluorescence. Images were taken at 200x magnification. (**D**) FACS analyses of the expression of F4/80 (FITC), CD86 (PE), and CD206 (PE/Cy7), markers of macrophages in M0, M1, and M2, respectively. Statistical analyses were performed using one-way ANOVA with Bonferroni’s multiple comparison post-test (* *p* ≤ 0.05; ** *p* ≤ 0.01; **** *p* < 0.0001).

PEMs represent tissue macrophages that could exhibit extraordinary plasticity, acquiring different functional phenotypes in response to the local microenvironment. Classically activated macrophages (M1) and alternatively activated macrophages (M2) are two extreme poles of this dynamic process known as macrophage polarization [58]. In particular, M1 macrophages are responsible for immune surveillance against pathogens, while M2 macrophages assume a predominant anti-inflammatory role by attenuating the host immune response. Interestingly, the phenotype of polarized M1 and M2 macrophages can also be achieved in in vitro experiments using specific cytokines and growth factors [59]. We thus investigated whether PEMs could be differentiated in vitro towards either the M1 or the M2 phenotypes. Considering the adherence properties of PEMs, they were isolated as described in the Materials and Methods section, and cultured for 5 days with the macrophage colony-stimulating factor (m-CSF) to promote their survival and proliferation into mature monocytes/macrophages [43,60]. Cells were then stimulated for 48 h with IFN-γ and LPS to promote M1 differentiation or treated with IL-4 to induce the M2 state (Figure 7A). To evaluate whether M1/M2 polarization in PEMs occurred, using q-RT-PCR, we analyzed the expression of some cytokines (TNF-α, COX-2, TGF-β), enzymes (iNOS, Arg-1), and the nuclear receptor PPAR-γ [61]. As shown in Figure 7B, the relative mRNA levels of iNOS, TNF-α, and COX-2 were significantly higher in macrophages treated with LPS/INF-γ (M1 phenotype), while the expression of Arg-1, TGF-β, and PPAR-γ was increased in macrophages treated with IL-4 (M2 phenotype). To better characterize the two phenotypes acquired in vitro by PEMs upon cytokine treatment, cells were stimulated as above, and the expression of specific markers for M1 and M2 phenotypes was analyzed by fluorescence microscopy. The positive staining of the M1 marker CD86 was visualized when cells were incubated with LPS and IFN-γ (Figure 7C, top line), whereas CD206 expression was significantly increased in PEM cells treated with IL-4 (Figure 7C, bottom line). These results were also confirmed by flow cytometry analysis. Indeed, Figure 7D shows that nearly 30% of F4/80^+^ cells expressed the mannose receptor CD206 on their surface, whose levels reached a value of 60% when PEMs were stimulated with IL-4. In contrast, the expression of CD206 decreased from 48% to 13% after LPS/IFN-γ treatment. In the mirror experiments, the 2% basal expression of CD86 protein was significantly increased to 48% following the stimulation with LPS/IFN-γ. Together, these findings indicate that PEMs can be induced to differentiate in vitro into M1 and M2 macrophages upon specific stimulation. Since the iron exposure of cells is correlated with the inflammatory macrophage phenotype [62,63], we also investigated the effects of PDA-MNPs on PEM polarization. Cells were cultured and then incubated several times with 100 μg/mL PDA-MNPs using macrophages treated with LPS/IFN-γ as a positive control. We found that the expression of genes associated with the pro-inflammatory phenotype was upregulated in PEMs after short incubations of PDA-MNPs (Figure S4A). In particular, the expression of iNOS was increased after 8 h of treatment with nanoparticles, while TNF-α and COX-2 levels were already elevated after 4 h of treatment. However, these genes were downregulated after 24 h of treatment, switching back to nearly basal levels. These data were also confirmed in other experiments, in which the expression of the M1 phenotype markers CD86 and CD80 was evaluated. Indeed, the expression of CD86 and CD80 was increased after 3 days of incubation with nanoparticles (Figure S4B). In particular, after PDA-MNP treatment, the expression of CD86 and CD80 in PEMs increased from 9% and 34% to 59% and 67%, respectively (Figure S4C). These experiments show that PDA-MNPs induce M1 macrophage polarization to a similar extent to what can be obtained by specific cytokine stimulation.

### 3.6. Reprogramming TAM-Like Macrophages toward the M1 Phenotype

**Figure 8:**
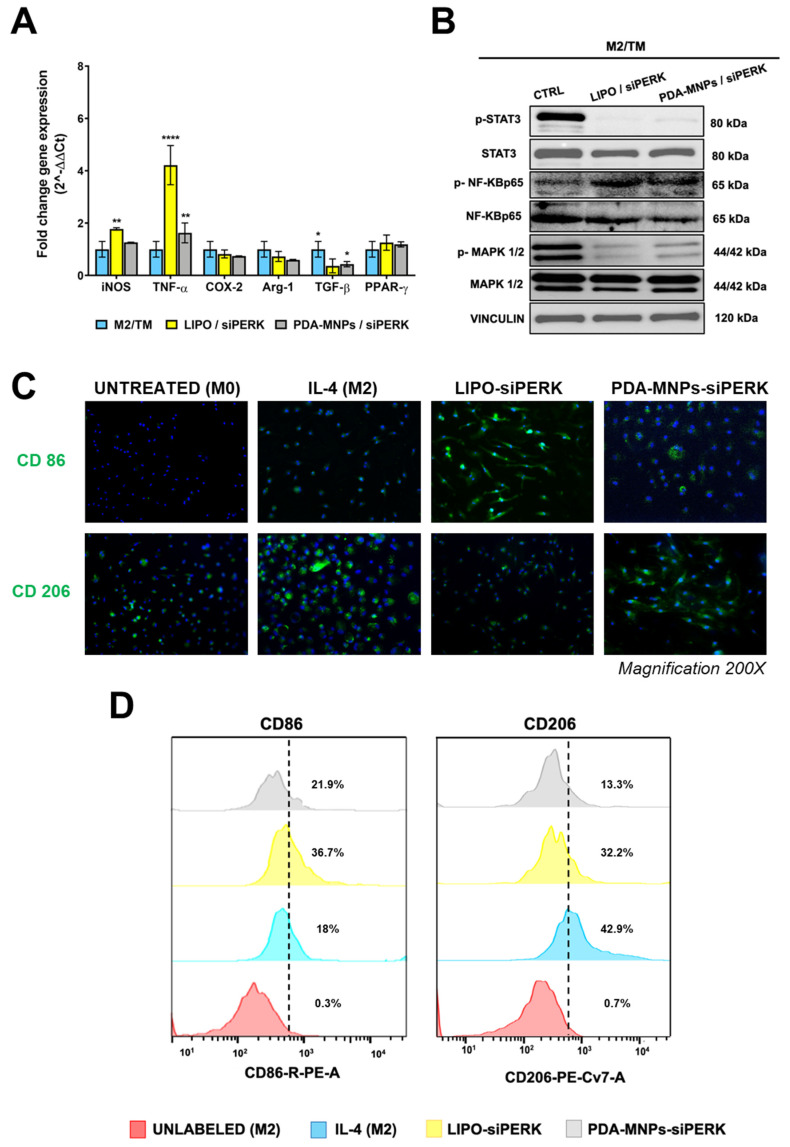
Reprogramming of TAMs from the M2 to the M1 phenotype. PEMs were polarized to the M2 phenotype with IL-4, treated with ER stress sensor tunicamycin (2 µg/mL), and incubated with LIPO/siPERK and PDA–MNPs/siPERK. (**A**) After 24 h of incubation, the mRNA levels of the genes specific for M1 or M2 phenotypes were determined by q–RT–PCR. (**B**) WB representing the expression and state of activation in M2/TM (TAM–like) of NF-*κ*B p65, STAT3, and MAPK1/2, 48 h after silencing PERK mRNA. Vinculin was used as the loading control. (**C**) Images showing the expression of CD86 (top row) and CD206 (bottom row) observed using fluorescence microscopy after M2/TM were incubated for 3 days with LIPO/siPERK and PDA–MNPs/siPERK, fixed, permeabilized, and stained with FITC–labeled antibodies and with DAPI (blue) for nuclei. Magnification: 200×. (**D**) Histograms showing the quantification of CD86 and CD206 expression determined by flow cytometry analysis after M2/TM were incubated for 3 days with LIPO/siPERK and PDA-MNPs/siPERK. The results for q–RT–PCR are expressed as the fold change ± SD with respect to the untreated M2/TM, obtained in three independent experiments made in triplicate and analyzed by one-way ANOVA with Bonferroni’s multiple comparison test (* *p* ≤ 0.05; ** *p* ≤ 0.01; **** *p* < 0.0001).

TAMs play a crucial role in the development and progression of many types of solid cancer due to their immunosuppressive activity [12]. It is well established that TAMs have a M2-like phenotype and display an irregular UPR response in their ER to endure the environmental stress to which they are subjected. Therefore, we performed a series of experiments to examine the ability of PEMs displaying a TAM-like phenotype to switch from the M2 to the M1 phenotype upon incubation with the PDA-MNPs/siPERK nanocomplexes, using LIPO/siPERK as an internal control. To obtain TAM-like macrophages, PEMs were polarized toward the M2 phenotype with IL-4, as previously described, and then treated with the ER stressor TM to activate the UPR response. Herein, PEMs will be named as M0—untreated, M1—treated with LPS/IFN-γ, M2—treated with IL-4, and M2/TM—treated with IL-4 and TM (TAM-like macrophage). In agreement with recent studies that reported that the PERK arm of the UPR is uniquely upregulated in TAMs [64,65], we validated this observation in our model, where the expression of PERK, as well as that of the downstream genes ATF4 and CHOP, were increased in PEMs treated with IL-4 and TM. We observed a significant inhibition of the PERK-eiF2α-ATF4-CHOP pathway when these cells were incubated with PDA-MNPs/siPERK or LIPO/siPERK (Figure S5A,B). Hence, PEM cells treated with IL-4 and TM represent a promising in vitro model to investigate the macrophage reprogramming of TAMs. For this reason, we evaluated the ability of PDA-MNPs/siPERK to re-educate these TAM-like macrophages from the M2 to the M1 phenotype by analyzing the expression of gene markers of the two phenotypes after the incubation with nanocomplexes, as well as with LIPO/siPERK. Notably, we found that in TAM-like macrophages, the downregulation of PERK enhanced the expression of M1 markers iNOS and TNF-α and decreased the expression of M2-specific genes such as Arg-1, TGF-β, and PPAR-γ (Figure 8A). In this scenario, we also investigated the effect of these nanocarriers on the transcriptional reprogramming of macrophages, which is a complex process regulated by different signaling molecular pathways, including NF-*κ*B, MAPKs, and STAT3 [66]. The activation of NF-*κ*B p65 is a hallmark of M1 macrophage activation, regulating the expression of an array of inflammatory genes; in addition, it is well known that its activation is associated with the overactivation of IRE1-α, the ER stress sensor that collaborates with PERK [67]. Instead, the activation of STAT3 drives the transcription of many genes associated with the M2 macrophage phenotype, typical of TAMs [68]. Likewise, ERK1/2 signaling activation facilitates macrophage differentiation toward the M2 phenotype, promoting angiogenesis, cancer cell migration, and invasion [69]. Thus, the phosphorylation levels of NF-*κ*B p65, STAT3, and ERK1/2 were analyzed after TAM-like macrophage (M2/TM) incubation with siRNA-lipo-complexes and nanocomplexes. The results showed that the downregulation of PERK in macrophages activated NF-*κ*B p65, while it inhibited the phosphorylation of STAT3 and ERK1/2 (Figure 8B and Figure S6), suggesting that the switch from the M2 to the M1 phenotype took place.

To confirm that PDA-MNPs/siPERK and lipo-complexes induced the re-polarization of TAM-like macrophages, the phenotype of these cells was examined at day 3 of treatment. As shown in Figure 8C, the expression of the CD206 mannose receptor on the surface of macrophages M2/TM decreased after the knockdown of the PERK protein, while the expression of CD86 increased. Indeed, Figure 8D shows that the expression of CD86 on the macrophage surface was significantly increased after the inhibition of PERK, particularly in the case of PDA-MNPs/siRNA. By contrast, the expression of CD206 decreased by about 30% after PDA MNPs/siPERK treatment. Together, these data indicate that TAM-like macrophages can be opportunely stimulated to switch to the M1-inflammatory phenotype by the inhibition of the ER stress sensor PERK.

## 4. Conclusions

We designed and tested a novel nanosystem to induce macrophage re-polarization from the pro-tumor M2 to the anti-tumor M1 phenotype by modulation of the UPR response in an experimental model of primary macrophages isolated from murine peritoneal exudate (PEMs). Herein, we describe how PDA-coated magnetic nanoparticles (MNPs) functionalized with siRNA molecules can target the UPR arm PERK, which is considered a critical metabolic hub for the immunosuppressive function of TAM-M2 macrophages. Results from the present study demonstrate that these nanoplatforms have good cytocompatibility and gene delivery capabilities into cells, which can be further increased by the application of an external magnetic field. In addition, PDA-MNPs/siPERK not only modulate the UPR in PEMs by inducing PERK gene silencing, but also affect the activity of other components of the UPR (XBP1s, ATF6). These biological effects, associated with both ER homeostasis and the immunomodulatory properties of PDA-MNPs, were able to restore the anti-tumor M1 phenotype of TAM-like macrophages. Therefore, these results show the suitability of these nanovectors for in vivo translational applications for targeted TAM immunotherapy in oncology and autoimmune diseases, where the macrophage balance is altered in the opposite way. Our results strengthen the availability of nanovectors for promoting useful biological effects, such as macrophage phenotype reconversion. Other authors have demonstrated the possible ability and in vivo compatibility of their models [12,25,28,32,33,35,41,48,49,62], and our experimental approach adds the possibility of exploiting the double opportunity, the advantages of magnetic guidance, and the modulation of gene expression. Our model has some critical issues that could be solved only with more accurate animal studies. Taken as a whole, our data and other authors’ models confirm that engineering nanomaterials might efficiently synergize traditional therapeutic treatments and open a wide range of possible applications in many fields of medicine.

## Figures and Tables

**Table 1:** Primer sequences for quantitative real-time PCR.

Target Gene	Forward Sequence	Reverse Sequence	Product Length (bp)
PERK	CCAGGCATTGTGAGGTATTT	TCTGTGCTTTCGTCTTTGAG	98
ATF4	GTTTAGAGCTAGGCAGTGAAG	CCTTTACACATGGAGGGATTAG	93
Chop	ACACGCACATCCCAAAG	ACCACTCTGTTTCCGTTTC	108
Bip	GAGAGAGGGAGAGAAGAACA	GCCACCACTTCAAAGACA	99
XBP1s	AGTCCGCAGCAGGTG	GGTCCAACTTGTCCAGAATG	93
ATF6	GGTCCAACTTGTCCAGAATG	TGGAGGTGGAGGCATATAA	109
Arg–I	ATCCCACCTAGGAGACAAAG	GGGACCTGGAATCTGTCTAT	116
TGF–β	CTCCCGTGGCTTCTAGTGC	GCCTTAGTTTGGACAGGATCTG	133
PPARγ	GTGACTCTGCTCAAGTATGG	GAACTCCCTGGTCATGAATC	114
iNOS	GTTCTCAGCCCAACAATACAAG	GTGGACGGGTCGATGTCAC	127
TNF–α	AGCCCCCAGTCTGTATCCTT	CTCCCTTTGCAGAACTCAGG	212
Cox–2	CTCCCTTTGCAGAACTCAGG	AGTGCTGGGCAAAGAATG	125
β-actina	GATGACCCAGATCATGTTTGA	GGAGAGCATAGCCCTCGTAG	161

**Table 2:** Antibody used for Western blot analysis.

Antigen	Species	Dilutions	Expected Band	Source	Cat. Number
PERK	Rabbit polyclonal	1/500	140 kDa	Cell Signaling Technology	C33E10
eif2–𝛼	Mouse monoclonal	1/500	38 kDa	Cell Signaling Technology	2103
Phospho eif2–𝛼	Rabbit polyclonal	1/500	38 kDa	Cell Signaling Technology	3398
STAT3	Mouse monoclonal	1/500	80 kDa	Cell Signaling Technology	9139
Phospho STAT3	Rabbit polyclonal	1/500	80 kDa	Cell Signaling Technology	9145
NF-*κ*B p65	Rabbit polyclonal	1/500	65 kDa	Santa Cruz Technology	sc-7151
Phospho NF-*κ*B p65	Mouse monoclonal	1/500	65 kDa	Santa Cruz Technology	sc-166748
MAPKK1/2	Rabbit polyclonal	1/500	42–44 kDa	Millipore	ABS44
Phospho MAPKK1/2	Rabbit polyclonal	1/500	42–44 kDa	Millipore	04-797
𝛼–Tubulin	Mouse monoclonal	1/500	55–60 kDa	Millipore	05-829
Vinculin	Mouse monoclonal	1/500	120 kDa	Santa Cruz Technology	sc-73614

**Table 3:** Primary antibodies used for immunofluorescence.

Antigen	Species	Dilutions	Source	Cat. Number
F4/80-BIOTIN	Mouse monoclonal	1/400	Miltenyi Biotec	130-116-514
CD206	Rabbit polyclonal	1/50	Abcam	ab64693
CD86	Rat	1/50	eBioscience	14-0862-82
CD80	Rat	1/50	eBioscience	553368

**Table 4:** Primary antibodies used for flow cytometry.

Antigen	Species	Dilutions	Source	Cat. Number
F4/80-FITC	Mouse monoclonal	1/100	eBioscience	11–4801–82
CD206–PE–Cy7	Mouse monoclonal	1/3600	eBioscience	25–2061–82
CD86–PE	Mouse monoclonal	1/100	eBioscience	12–0862–82
CD80–PE	Mouse monoclonal	1/100	eBioscience	16–10A1
CD11b–FITC	Mouse monoclonal	1/100	eBioscience	11–0112–82
CD11b–PE	Mouse monoclonal	1/100	eBioscience	12–0112–83
CD11b-PE-Cy7	Mouse monoclonal	1/100	eBioscience	25–0112–82
CD45–APC–eFluor 780	Mouse monoclonal	1/100	eBioscience	47–0451–82
CD3 FITC	Mouse monoclonal	1/50	eBioscience	11–0032–82
CD19 APC	Mouse monoclonal	1/50	eBioscience	17–0193–82
Ly–6G/Ly–6C (Gr1)PerCP-Cyanine 5.5	Mouse monoclonal	1/300	eBioscience	45-5931–80

## Data Availability

Not applicable.

## Supplementary Materials

The following supporting information can be downloaded at: https://www.mdpi.com/article/10.3390/pharmaceutics15061711/s1, Figure S1: Degradation of PDA coated on MNPs and release of siRNA coupled to PDA-MNPs; Figure S2: Cytocompatibility of PDA-MNPs in RAW 264.7 cells; Figure S3: Interaction of PDA-MNPs with RAW 264.7 cells in the presence/absence of a gradient magnetic field (GMF); Figure S4: The effects of PDA-MNPs on the macrophage polarization of PEMs; Figure S5: Inhibition of the PERK pathway by LIPO/siPERK and PDA-MNPs/siPERK in TAM-like macrophages; Figure S6: Densitometric analysis of the bands of STAT3, NF-*κ*B p65, and MAPK1/2; Figure S7: Uncropped agarose gels and Western blot; Table S1: Properties of the nanocomplexes.
