# Supplementary material for: Macrophage Reprogramming via the Modulation of Unfolded Protein Response with siRNA-Loaded Magnetic Nanoparticles in a TAM-like Experimental Model
Source: Pharmaceutics. 2023 Jun 12;15(6):1711. doi: 10.3390/pharmaceutics15061711 (PMC10302170; doi:10.3390/pharmaceutics15061711)
Supplement: Supplementary file 1 [file pharmaceutics-15-01711-s001.zip › pharmaceutics-2403562-supplementary.pdf]

Macrophage Reprogramming via the Modulation of Unfolded Protein Response with siRNA-Loaded Magnetic Nanoparticles in a TAM-like Experimental Model

Annarita D'Urso , Francesca Oltolina, Chiara Borsotti, Maria Prat, Donato Colangelo * and Antonia Follenzi *

Table S1. Properties of the nanocomplexes.

Sample	Color	DLS (nm)	Zeta potential (mV)
MNPs	Dark Brown	15 ± 8 nm	0.65
PDA-MNPs	Black	30 ± 5 nm	-0.2
PDA-MNPs/siRNA	Black	42 ± 1. 73	-5.3

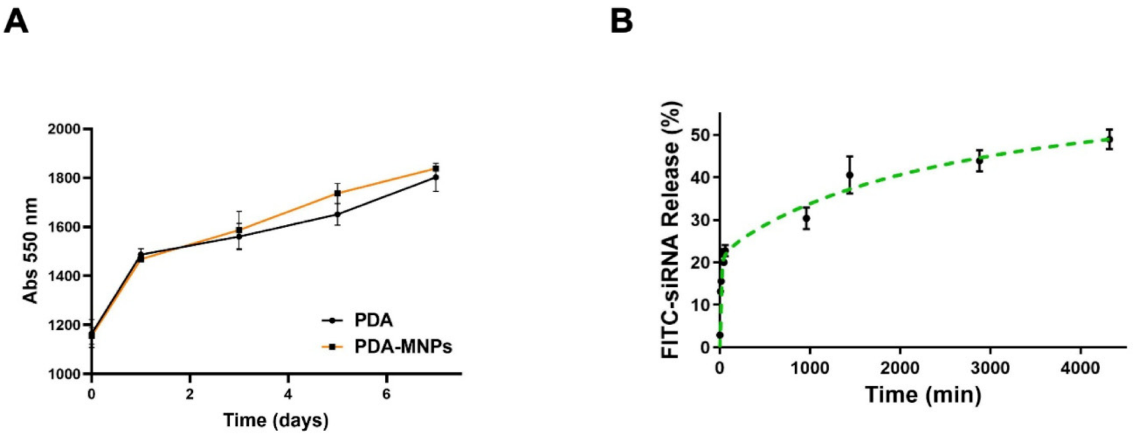

Figure S1. Degradation of PDA coated on MNPs and release of siRNA coupled to PDA-MNPs. (A) Absorbance values of PDA coupled to MNPs obtained from the supernatants derived by magnetic decantation during different time points. Values of PDA obtained by dopamine polymerization in absence of MNPs was used as reference value. **(B)** Release behaviour of FITC-siRNA adsorbed on PDA-MNPs performed in complete medium at 37° and at different time points.

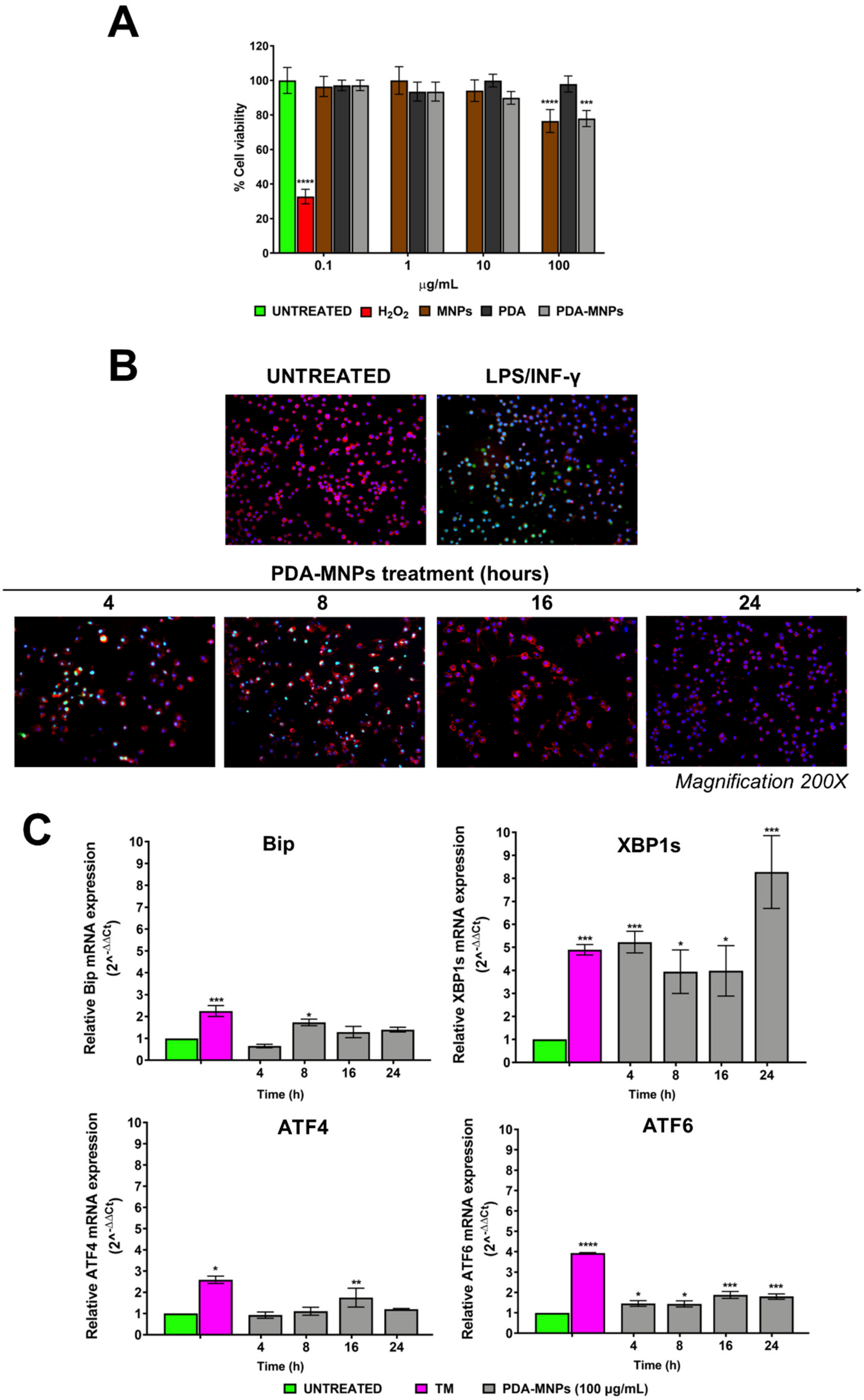

Figure S2. Cytocompatibility of PDA-MNPs on RAW 264.7cells. (**A**) Cell viability was assessed with MTT assays after cells were incubated with PDA-MNPs at different concentrations for 72 h. Untreated cells were taken as reference value

(100%), while the exposure to H_2O_2 at 1 μM represented the positive control. Data are expressed as means ± SD of at least four independent experiments performed in triplicate. (**B)** Immunofluorescence images showing reactive oxygen species (ROS) production in RAW 264.7 cells incubated with PDA-MNPs (100 μg/mL) for different time points (from 4 to 24 h). Fixed and permeabilized cells were stained for actin with TRITC-phalloidin (red) and for nuclei with DAPI (blue), while the ROS production was visualized in green. LPS/INF-γ 24 h treated cells were used as positive control. Magnification, 200x. (**C)** Graphs showing the expression of ER-stress markers (Bip, XBP1s, ATF4 and ATF6) assessed by q-RT-PCR on RAW 264.7 cells incubated with PDA-MNPs (100 μg/mL), in the same conditions as above. ER-stress was mostly observed in cells treated with tunicamycin 2μg/mL for 8 h, used as positive control. Gene expression is shown as fold-change relative to untreated cells. Data are expressed as mean ± SD of three independent experiments. Statistical analyses were carried out using One-way ANOVA, with Bonferroni comparison post-test vs the untreated controls (* $p \leq 0.05$; ** $p \leq 0.01$; *** $p \leq 0.001$; **** $p < 0.0001$).

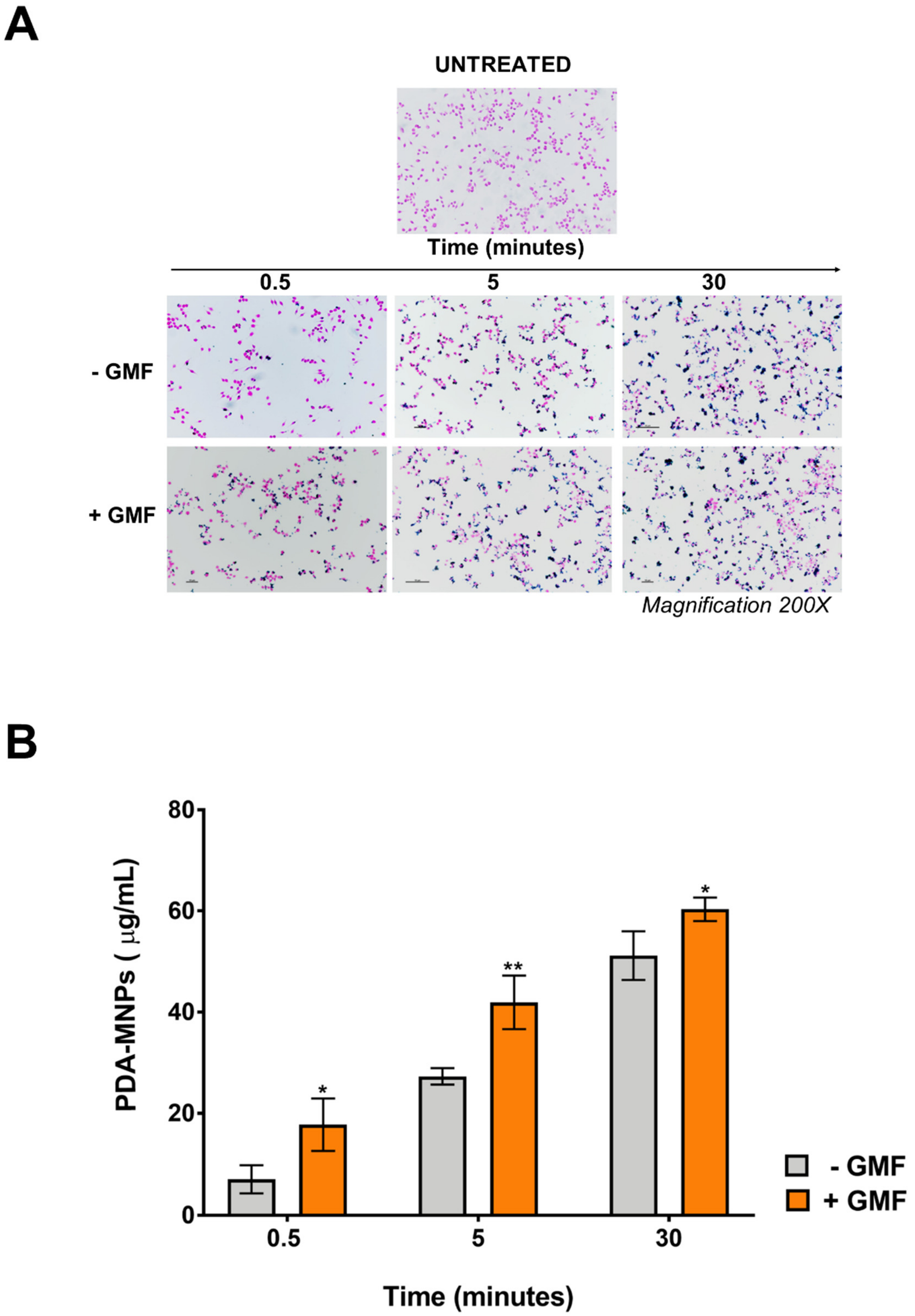

Figure S3. Interaction of PDA-MNPs with RAW 264.7cells in presence/absence of a gradient magnetic field (GMF). (A) Images show RAW 264.7 cells treated with PDA-MNPs (100 µg/mL) and stained Prussian blue staining and nuclear fast red. Magnification 200x. **(B)** Amount of iron associated with cells as quantified with potassium thiocyanate: cells were incubated with PDA-MNPs for different time-points (from 0.5 to 30 min) in absence (-GMF) or in presence (+GMF) of a gradient magnetic field. Untreated cells were used as negative control. Results are expressed as mean µg/mL ± SD, and were obtained from at least three independent experiments performed in triplicates. Data were analyzed by t-test for each time point. (* $p < 0.05$; ** $p < 0.01$).

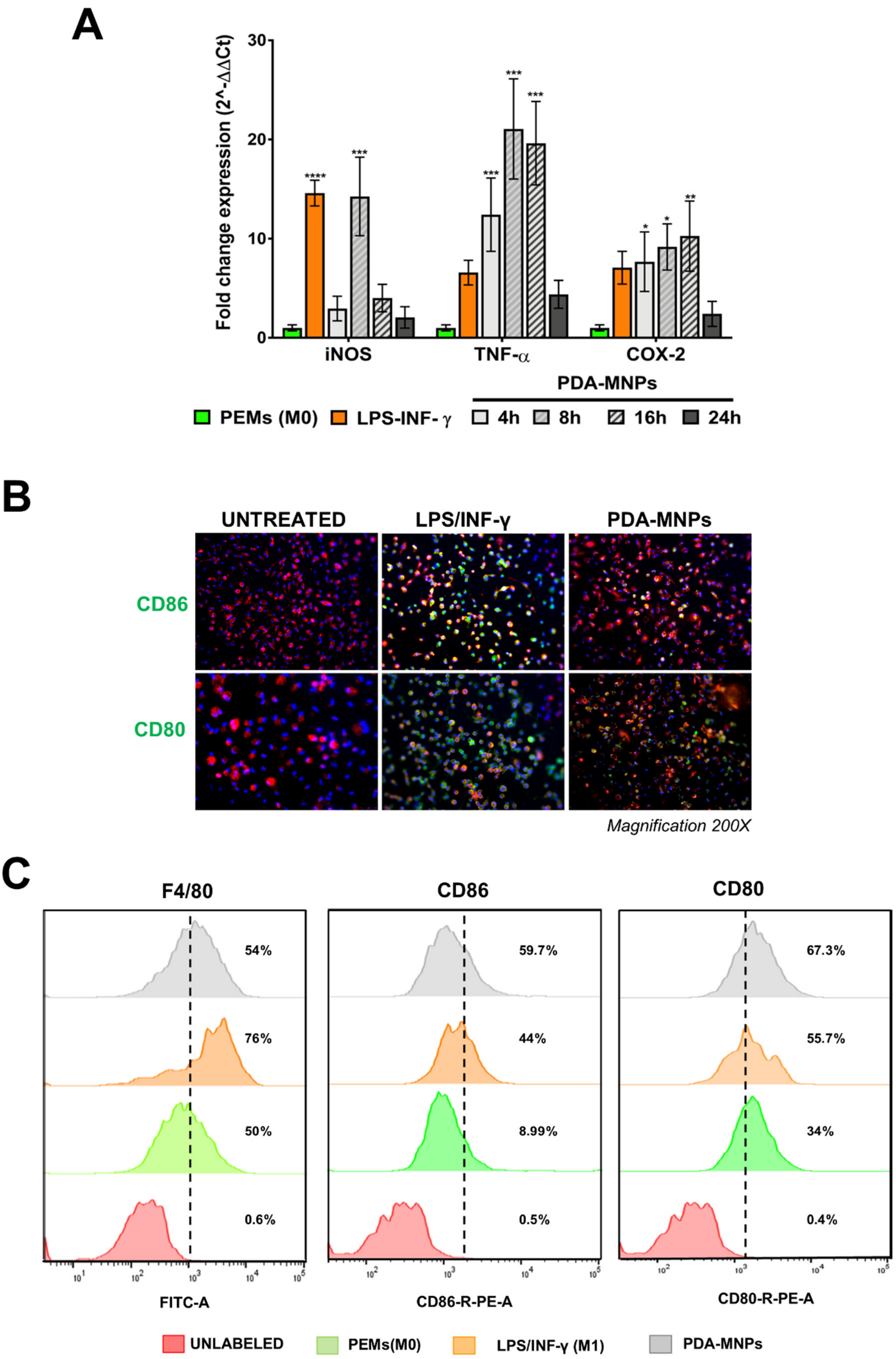

Figure S4. The effects of PDA-MNPs on the macrophage polarization of PEMs. (A) Relative expression of specific genes for M1 phenotype was determined by q-RT-PCR after cellular incubation with PDA-MNPs (100 μg/mL) for different time points. M0 cells (green) and LPS/ IFN-γ induced-PEMs (M1) (orange) were used as controls, and gene expression was

expressed as fold-change relative to M0 cells. Experiments were performed in triplicates; data represent fold change ± SD of three experiments. **(B)** PEMs were incubated with PDA-MNPs (100 μg/mL) for 3 days and the expression of CD86 and CD80 was visualized at fluorescence microscopy. Macrophages are visualized in red (FITC-Streptavidin-F4/80), nuclei in blue (DAPI), while the expression of CD86/CD80 are visualized as the green dots. Magnification 200x. **(C)** Cells were incubated with PDA-MNPs (100 μg/mL) for 3 days and then, the percentage values of CD86 and CD80 expression were assessed by flow cytometry. Unlabeled M0 were used to set-up the measures in flow cytometry experiments. Statistical analyses were performed using One-Way Anova followed by Bonferroni 's post-test (* $p \leq 0.05$; ** $p \leq 0.01$; *** $p \leq 0.001$; **** $p < 0.0001$).

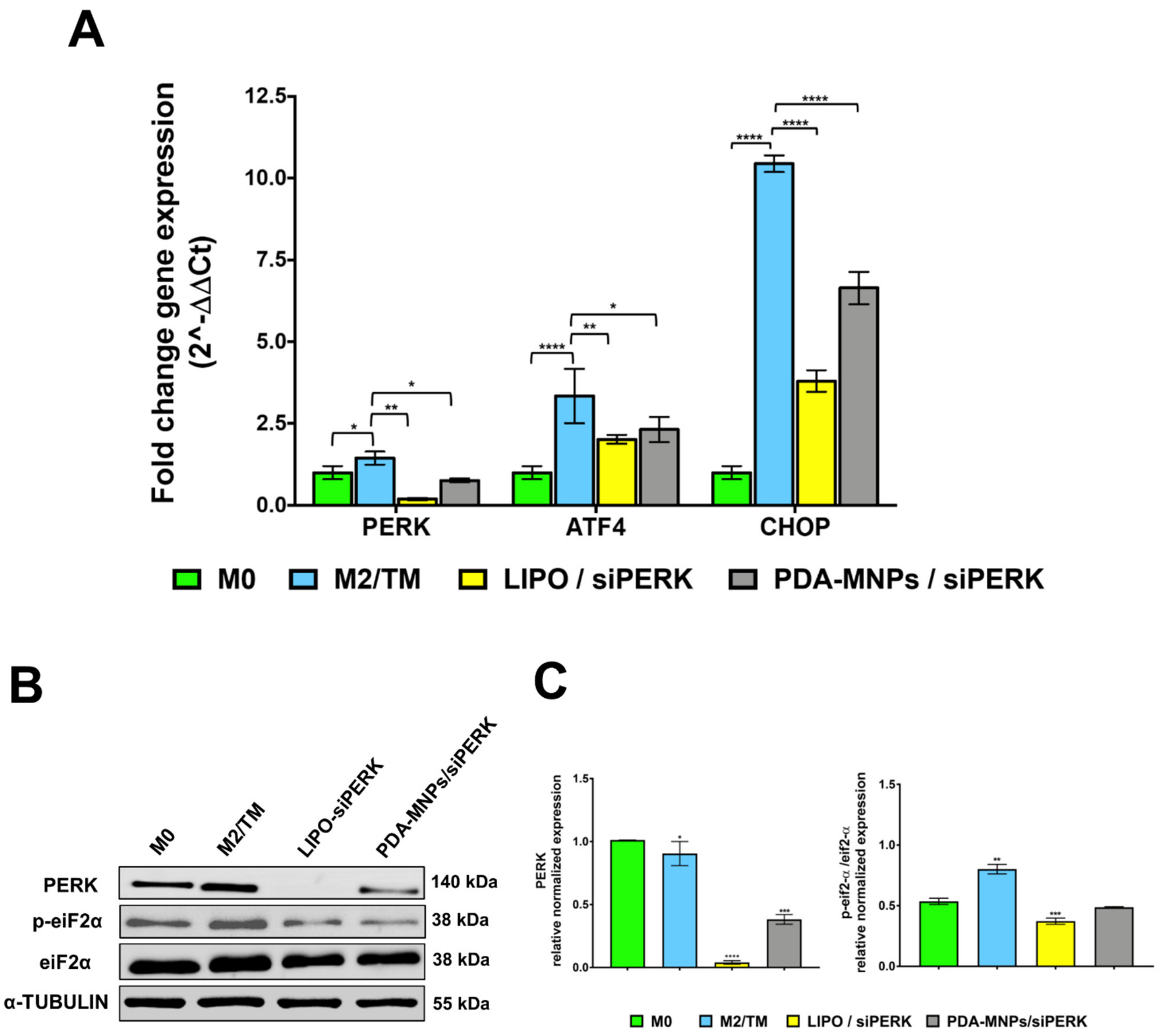

Figure S5. Inhibition of PERK pathway by LIPO/siPERK and PDA-MNPs/siPERK in TAMs-like macrophages. PEMs were polarized to M2 phenotype with IL-4, treated with ER-stress sensor tunicamycin (2μg/mL) and incubated with LIPO/siPERK, PDA-MNPs/siPERK. **(A)** After 24h incubation the mRNA levels of PERK, ATF4, CHOP were assessed by q-RT-PCR. Gene expression was expressed as fold-change relative to the untreated M2/TM. **(B)** After 48h the expression of PERK and the state of phosphorylation of Eif2-α was analyzed by immunoblot assay and **(C)** the densitometric analysis of protein bands was shown using the expression of α-Tubulin as internal control. Data are expressed as the mean ± SD of three independent experiments and analyzed by One-way ANOVA with Bonferroni's multiple comparison post-test (* $p \leq 0.05$; ** $p \leq 0.01$; *** $p \leq 0.001$; **** $p < 0.0001$).

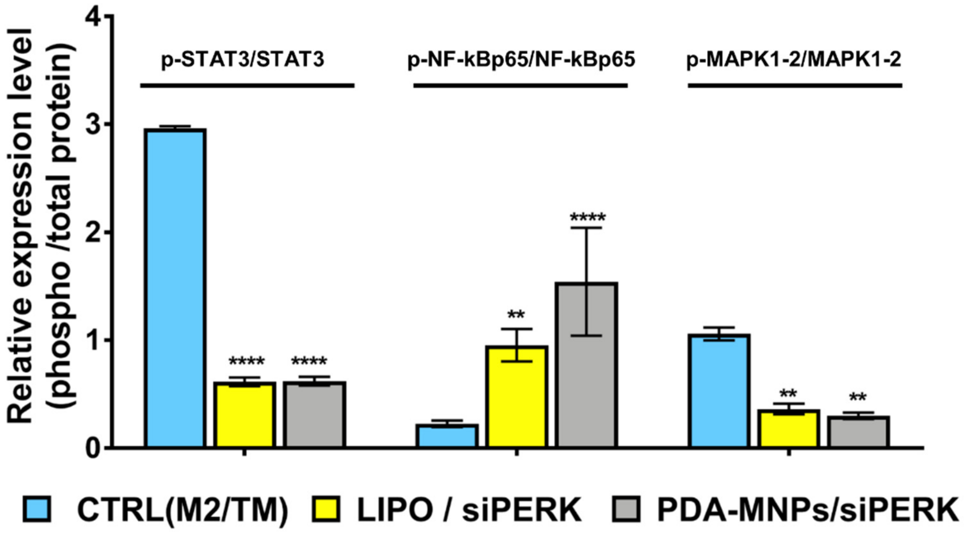

Figure S6. Densitometric analysis of bands of STAT3, NF-kB p65 and MAPK1/2. Histograms show the relative densitometric analysis of the bands of STAT3, NF-kB p65 and MAPK1/2, 48h after silencing PERK in PEMs (M2/TM). Vinculin was used as internal control using the ImageJ software. Data are expressed as the mean ± SD of three independent experiments and analyzed by One-way ANOVA with Bonferroni's multiple comparison post-test (** $p \leq 0.01$; **** $p < 0.0001$).

ORIGINAL IMAGES FOR GELS AND BLOTS

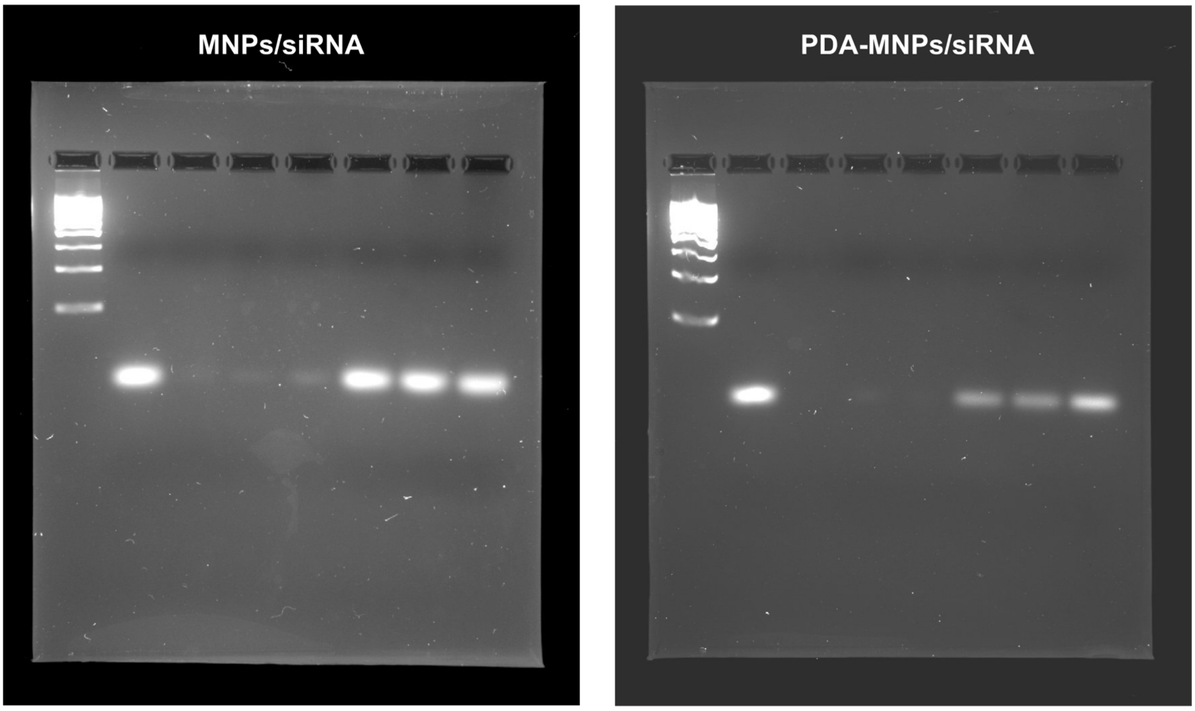

Original images of agarose gels showing the loading of siRNA onto MNPs and PDA-MNPs along with unbound siRNA left in their supernatants after the magnetic decantation. The first band following MW shows the naked siRNA used as control in both images. In the manuscript these representative images were shown in Figure 5A.

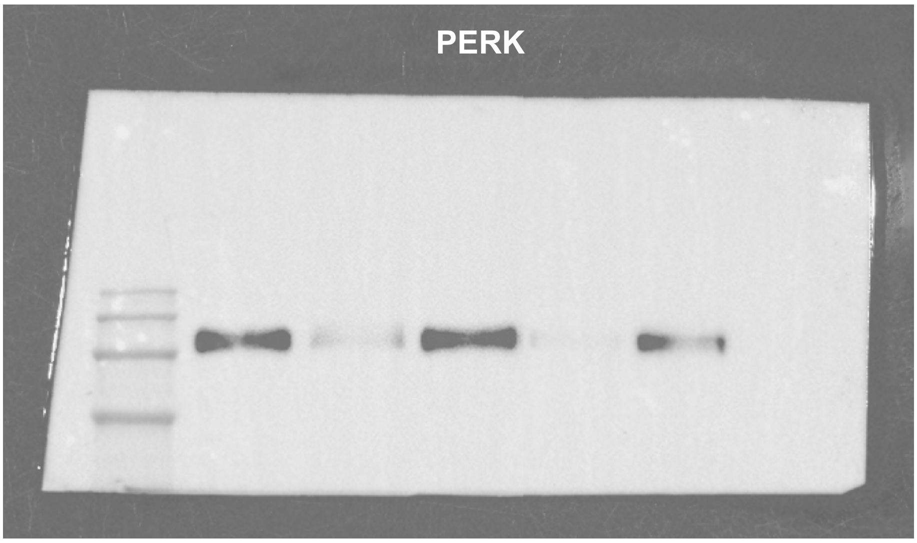

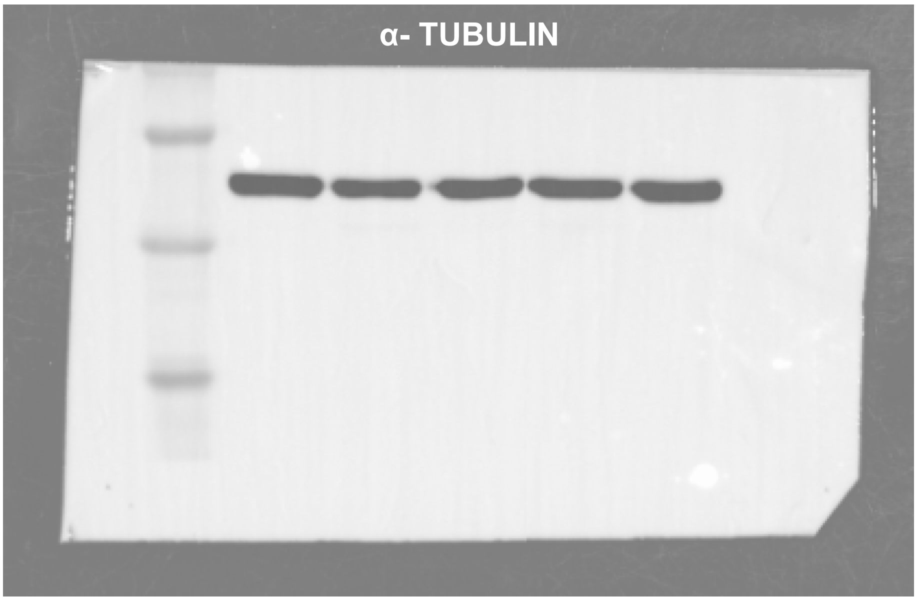

Original Western Blots relative to the gene silencing of PERK protein in PEMs with the relative control α-Tubulin. This α-Tubulin image was used as reference for all the Western blots shown in Figure 6A of the manuscript.

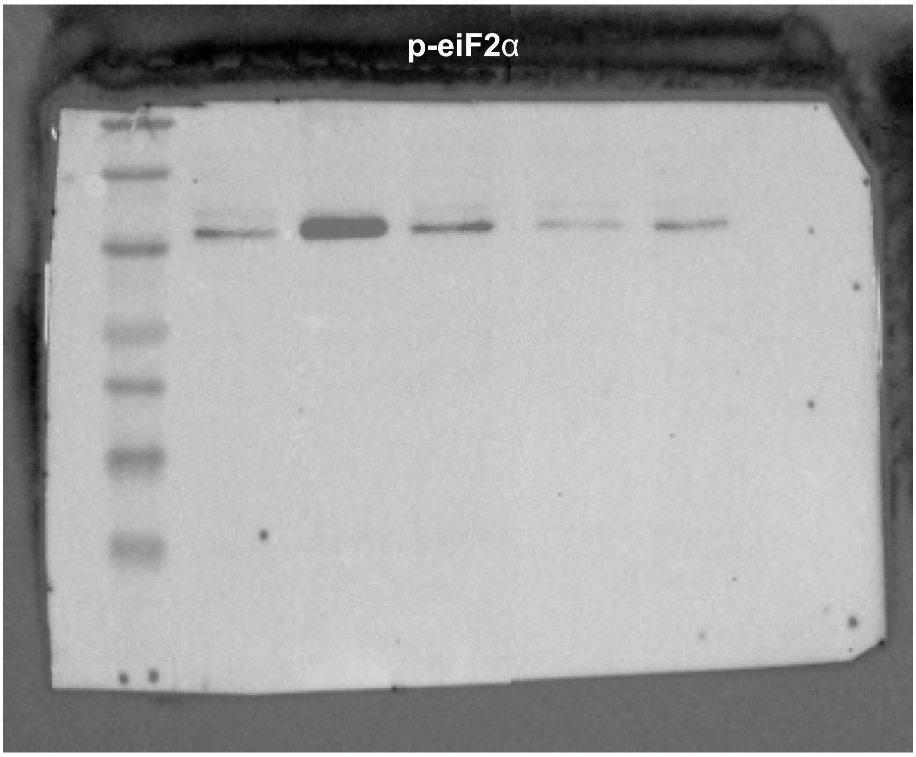

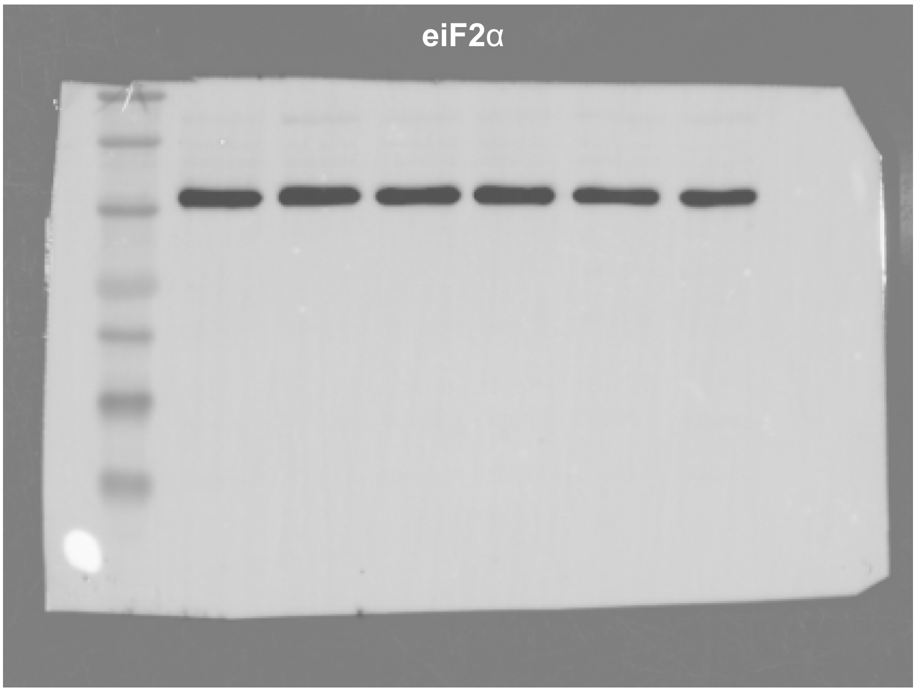

Other Western blots shown in the Figure 6A of this manuscript. The PVDF paper was first incubated for the visualization of the phosphorylated form; then incubated again for the total form of the protein.

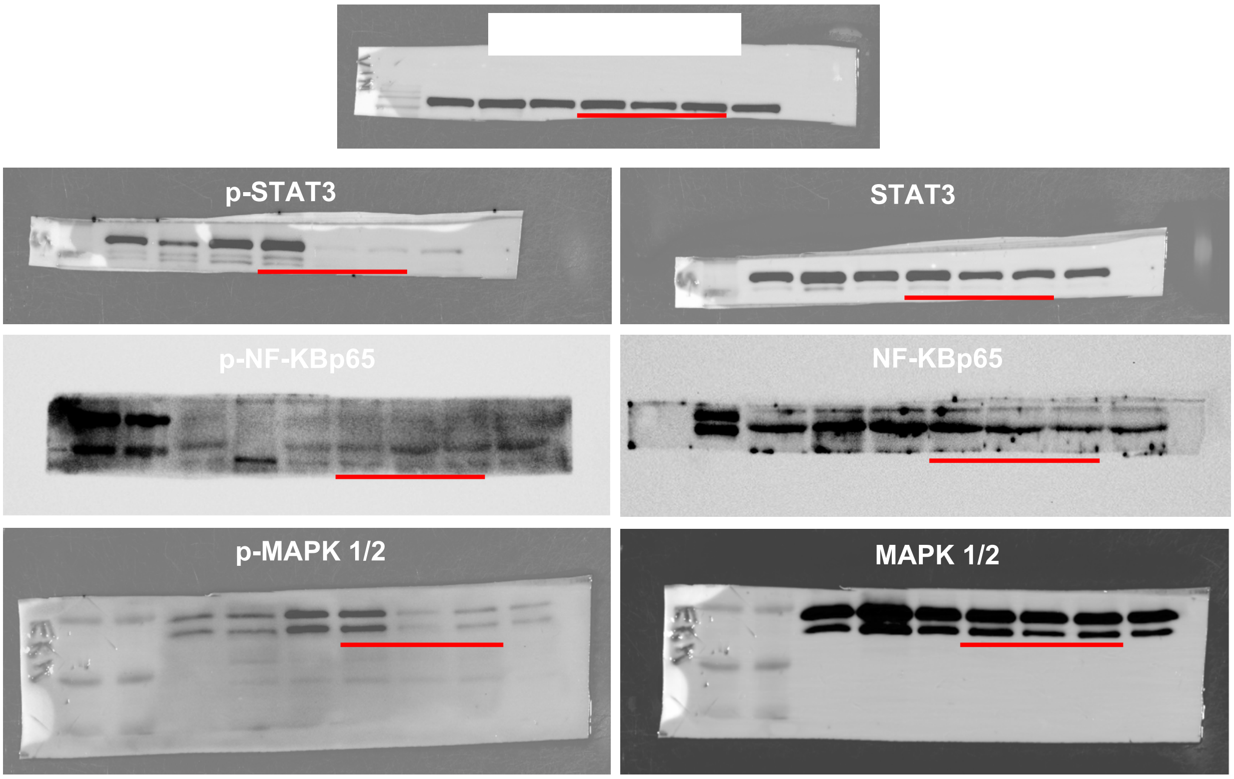

For each target the PVDF paper was first incubated for the visualization of the phosphorylated form; then stripped and incubated again for the total form of the protein. A representative WB for VINCULIN performed from the same extracts run for the analysis of other proteins was shown. The red lines refer to the three lanes reported in the Figure 8B of this manuscript.

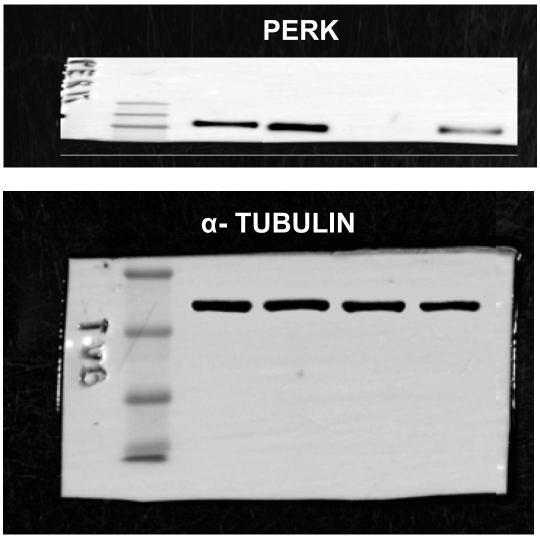

Original Western Blots relative to the gene silencing of PERK protein in PEMs (M2/TM) with the relative control α-Tubulin. This α-Tubulin image was used as reference for all the Western blots shown in Figure S4B in the supplementary of the manuscript.

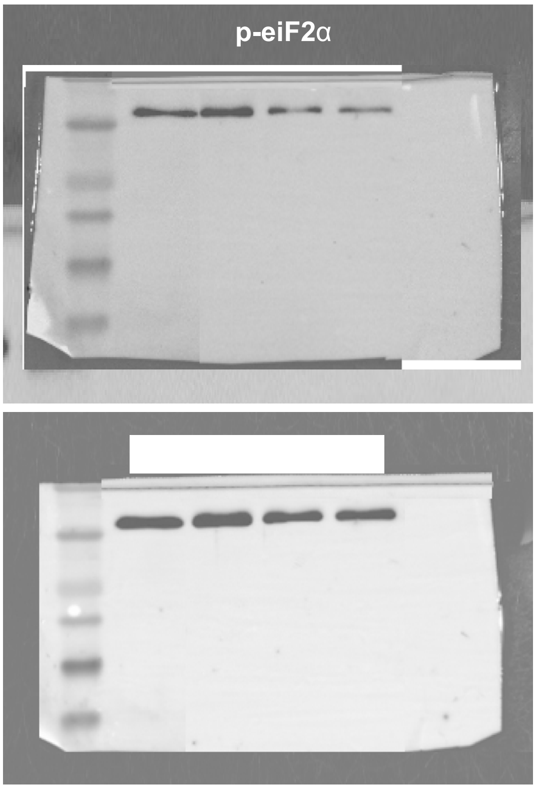

Other Western blots shown in the Figure S4B of the supplementary. The PVDF paper was first incubated for the visualization of the phosphorylated form; then incubated again for the total form of the protein.

Figure S7. Uncropped agarose gels and Western blot.
